# The Complex Relationship Between High Temperatures and Avian Breeding Success: Insights From a Global Review

**DOI:** 10.1002/ece3.71771

**Published:** 2025-07-28

**Authors:** Adrien Levillain, Sophie Reichert, Sylvie Massemin

**Affiliations:** ^1^ CNRS, IPHC UMR 7178 Université de Strasbourg Strasbourg France; ^2^ Department of Biology University of Turku Turku Finland

**Keywords:** avian, breeding success, climate warming, high temperature, phenology, reproduction

## Abstract

Climate change is one of the major threats to biodiversity. Understanding how species cope with increasing temperature is of prime importance when assessing population viability. We present a systematic review of the association between high temperature and the breeding success of wild birds. We focus on avian species, as they are widespread throughout the world and benefit from numerous long‐term monitoring programs. We conducted a survey in the Web of Science library and retained 229 studies based on our eligibility criteria. We qualitatively assessed whether studies investigated the effect of high temperatures. High temperatures were defined in regard to the average temperature recorded at the study site. The species thermoregulation was taken into account depending on the information available. We were able to extract the local climate type (i.e., arid, temperate, continental, polar, and tropical) for 135 studies. Temperate and continental climates were over‐represented, and studies were more likely to investigate the relationship between hot events and breeding success in arid environments. The relationship between high temperatures and breeding success is highly complex, as it most likely involves a combination of “direct” effects (mediated through thermoregulation) and “indirect” effects (mediated through phenology, food availability, trophic interactions) and may vary depending on the system studied. Finally, we present some considerations for future studies, in particular regarding species' sensitivity at high temperatures.

## Graphical abstract

This article is a systematic review of wild birds breeding success under high temperatures. We highlight the influence of the local climate and emphasize the need to disentangle direct (i.e., through thermoregulation) and indirect effects (i.e., through phenology or trophic relationship).

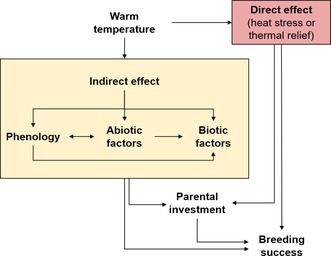

## 1 Introduction

Climate change emerges as one of the main drivers of the current biodiversity erosion (Maxwell et al. 2016). The main environmental changes associated with climate change are global warming and an increase in the frequency of extreme stochastic events such as heatwaves, which are predicted to gain in frequency, intensity, and duration over time (IPCC 2014; Meehl and Tebaldi 2004; Stillman 2019; Ummenhofer and Meehl 2017). Climate change may impact diverse populations through varying mechanisms such as variation in species demography (Paniw et al. 2021), phenology (Cohen et al. 2018; Neate‐Clegg et al. 2024), and ranges of distribution (Devictor et al. 2008; Kubelka et al. 2022; Pacifici et al. 2020). These observations can find an explanation either through direct variation in the thermal environment (e.g., Albright et al. 2017; Conradie et al. 2020) or indirectly through changes in trophic relationships (e.g., DeGregorio et al. 2015; Pearce‐Higgins and Morris 2023).

From a thermoregulation standpoint and for endotherms, the terms “high temperature” usually refers to temperatures promoting heat stress, which can be defined as a temperature that is eliciting behavioral and physiological adjustments to maintain body temperature and/or water homeostasis and is most commonly represented by the upper critical temperature (UCT, see glossary; McKechnie and Wolf 2019). In ecological studies, different authors refer to “high temperature” as relative to the species sensitivity (e.g., thermoregulatory response and fitness costs) or to the local climate variability. Defining “high temperatures” in an ecophysiological context is a real challenge, especially when focusing on a global scale. First, studies that vary widely use their use of temperature variables and statistical procedure (e.g., average of daily mean temperature, number of days exceeding a threshold…) and do not always report temperature data or summary statistics. Moreover, temperature variables are often computed on a large period of time, consequently buffering extreme values. Secondly, field studies most commonly use air temperature, which do not equate to the environmental temperature (i.e., ambient or operative temperature in laboratory and field settings respectively), further limiting the relevance of a direct comparison with species thermoneutral zone (TNZ; Mitchell et al. 2024). Additionally, TNZ has limited predictability when considering *in natura* scenarios, and thermal limits for breeding performance may differs from UCT (Clusella‐Trullas et al. 2021; MacMillan 2019; Mitchell et al. 2018). Finally, species TNZ are not systematically known, especially for polar birds. In this review, we defined “high temperature” as above average temperature for the system studied, or relative to the species thermoregulation depending on the information available (See methods). We qualitatively assessed whether studies investigated “high temperature” as “hot events” or “above average temperature”.

Birds represent relevant sentinel species for global changes. They exploit habitats ranging from deserts to polar biomes all around the globe and benefit from a multitude of long‐term monitoring programs. Warming temperatures have been linked with decreasing abundance (Iknayan and Beissinger 2018; Milne et al. 2015; Riddell et al. 2019) and heatwaves with mass‐mortality events (McKechnie, Gerson, et al. 2021; Piatt et al. 2020) and complete breeding failures (McCowan and Griffith 2021; Romano et al. 2020; Sharpe et al. 2021) of various bird species. Overall, reproductive success is a key factor when assessing avian species population viability, and temperature seems to be an important mediator of reproductive success for multiple populations across the globe (e.g., Jenouvrier et al. 2003; Chase et al. 2005; Kentie et al. 2018; Jansen et al. 2019). Summarizing how birds respond to the thermal environment is a complex task, and past reviews have focused on thermoregulation (Boyles et al. 2011; Cunningham et al. 2021), parental care (Du and Shine 2015; Mainwaring et al. 2016; Durant et al. 2019; Andreasson, Hegemann, et al. 2020), growth and development (Hepp et al. 2015; Nord and Giroud 2020; Sauve et al. 2021), and phenology (Jones and Cresswell 2010; Møller et al. 2010). More recently, a meta‐analysis reported that avian breeding success may decrease in warming areas (Halupka et al. 2023). We present here a systematic review of wild birds' breeding success under high temperatures.

**FIGURE 1:**
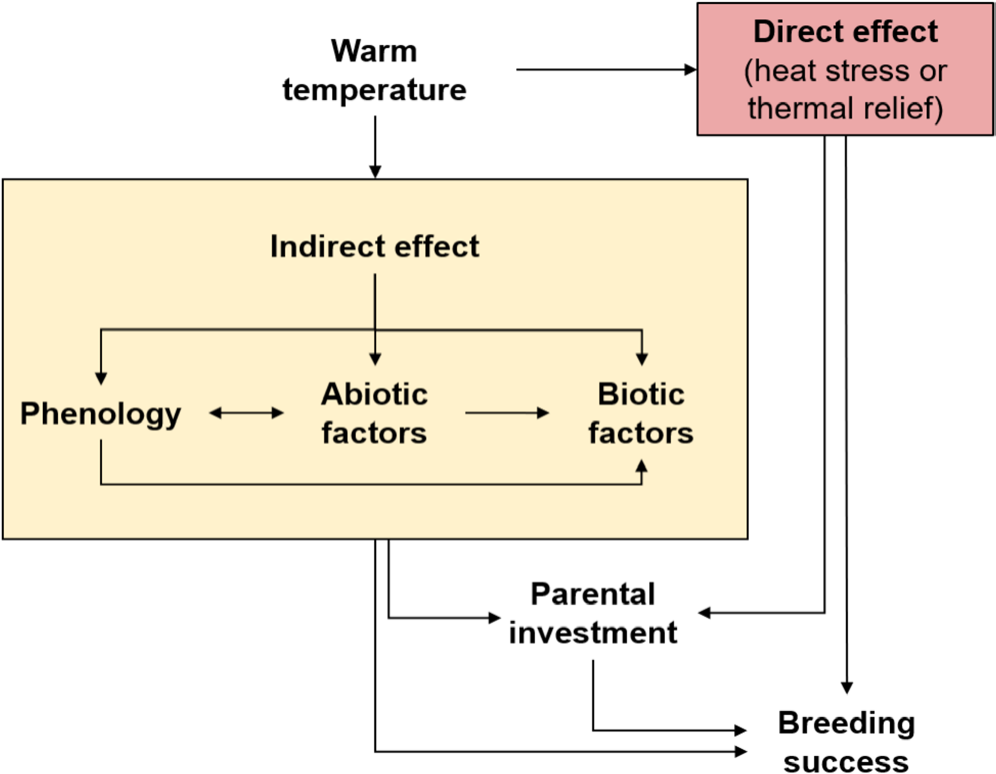
Schematic representation of the mechanisms that potentially mediate the link between high temperature and breeding success. High temperature can influence breeding success through direct (i.e., via thermoregulation of parents and offspring) or indirect ways such as variation in phenology (e.g., breeding timing and breeding season duration), abiotic factors (e.g., snow coverage and water availability) or biotic factors (e.g., food availability, predation, parasitism, and competition).

In this review, we aim to highlight potential trends and mechanisms that link avian breeding success and high temperatures (Figure 1). Breeding success can be directly driven by the environmental temperature (i.e., mediated via parents or offspring themoregulation; e.g., McCowan and Griffith 2021; van de Ven et al. 2020). However, the link between breeding success and temperature can be more elusive and may rely on other mechanisms than thermoregulation alone (e.g., Antoniazzi et al. 2011; D'Amelio et al. 2022; Vatka et al. 2011). In this review, we discuss « direct » effects of high temperature, that is mediated via thermoregulation of parents or offspring. We most specifically provide an overview of how heat stress or a thermal relief (i.e., can drive avian breeding success) when exposed to high temperatures (i.e., decreased energy needed to maintain nest or body temperature through thermogenesis). We then provide an overview of potential indirect effects of high temperatures, such as mediated by variation in phenology (e.g., breeding timing and duration of the breeding season), abiotic factors (e.g., snow coverage and water availability) or trophic interactions (e.g., food availability, predation, parasitism, and competition). Finally, we briefly present perspectives and considerations for future studies, ranging from experimental design to data analysis and insist on the importance of disentangling relationships within a system.

## 2 Material and Methods

### 2.1 Literature Survey

We searched in ISI Web of Science library on 01/13/2022, using the keywords “((ALL=“avian$” OR ALL=“bird$”) AND (TS=“high* temperature$” OR TS=“warm* temperature$” OR TS=“increas* temperature$” OR TS=“warm*” OR TS=“heat*” OR TS=“hot”) AND (TS=“breeding” OR TS=“reproduct*” OR TS=“fledg*” OR TS=“chick$” OR TS=“hatch*” OR TS=“nest*”) AND (TS=“success” OR TS=“surviv*” OR TS=“mortality” OR TS=“productivity” OR TS=“recruitment” OR TS=“clutch size”) NOT (ALL=“poultry” OR TS=“broiler“))”.

We only retained studies that met our eligibility criteria, that is, studies that simultaneously (1) investigated the relationship between high temperature during the breeding season and breeding success *in natura*, meaning we excluded thermal manipulation experiments or captive breeding studies; (2) considered the local air temperature, that is, studies on nest microclimate or sea surface temperature were not retained to limit the heterogeneity of approaches; (3) described temperature using the keywords “warm(er)”, “hot(ter)”, “high(er)”, “increasing”, “heat”, or “heatwave”. Monitoring programs spanning more than 10 years were considered to have sufficient temperature variation and were automatically retained; (4) reporting results per species (i.e., either studies focusing on a single species, or report statistical analyses separately for each species when investigating multiple species). In the latter case, data for each species were considered independent and are hereafter referred to as “study”; and (5) do not include data duplication. When the same dataset was shared by two articles, they were merged as a single study in our database. The literature search yielded 1214 results ranging from year 1990 to 2022 (Data S1). We retained 438 articles upon abstract reading and 118 after full‐text inspection for eligibility criteria. We added 37 articles upon reading the selected articles, resulting in a total of 155 articles. After checking all articles for single species analysis, we ended up with a total of 229 studies (Data S2).

We defined five stages of breeding success: S0 (breeding propensity; probability of breeding, nesting probability, nest occupancy, nest density), S1 (clutch size), S2 (Hatching success; proportion or number of eggs hatching, probability of producing at least one hatchling, daily nest survival), S3 (post‐hatching success; proportion or number of hatchling‐nestling reaching the nestling‐fledgling stage, probability of producing at least one nestling‐fledgling, daily nest survival, young to adults ratio), and S4 (post‐fledging success; winter‐first‐year recapture probability, recruitment rate). The daily survival rate computed from the egg stage to the post‐hatching stage was considered in the stage S3.

### 2.2 Definition of High Temperature

We defined “high temperature” as relative to the average temperature at the study site, or relative to the species thermoregulation. We qualitatively assessed whether the retained studies investigated either (A) “hot events”: (A1) *T*_*a*_ likely to promote heat stress (T_a_ > UCT, behavioral signs of heat dissipation, signs of dehydration or hyperthermia), (A2) extreme events according to local climatic variability (e.g., 90th percentile of the hottest days), (A3) according to authors description (e.g., described as “heatwave”); or (B) “above average temperature”: (B1) according to local the local climatic variability (e.g., significant variation in temperature between years), (B2) according to authors description (e.g., “high” or “warm temperature”), (B3) above average temperature assumed to be investigated for long‐term studies (≥ 10 years of monitoring).

### 2.3 Mapping Study Location and Data Acquisition

The locations of the studies were extracted directly from the coordinates or based on the authors' description. No coordinates were extracted for studies investigating multiple sites further than 100 km, or with a range exceeding 5000 km^2^. These thresholds were chosen to remove studies that could potentially span multiple climates from the analysis. We obtained the study location for 135 of a total of 229 studies. We extracted the local climate at each study site from the Köppen‐Geiger climate classification map (0.0083° resolution, main climate at 0.5° diameter; Beck et al. 2018). The Köppen‐Geiger climate classification distinguishes 31 climate types based on local precipitation and temperature and regroup them into the 5 main climate groups: “Tropical”, “Arid”, “Temperate”, “Continental”, and “Polar” All spatial data treatment were conducted in QGIS (Version 3.16.16).

## 3 Overview of the Studies

### 3.1 System and Species Studied

**FIGURE 2:**
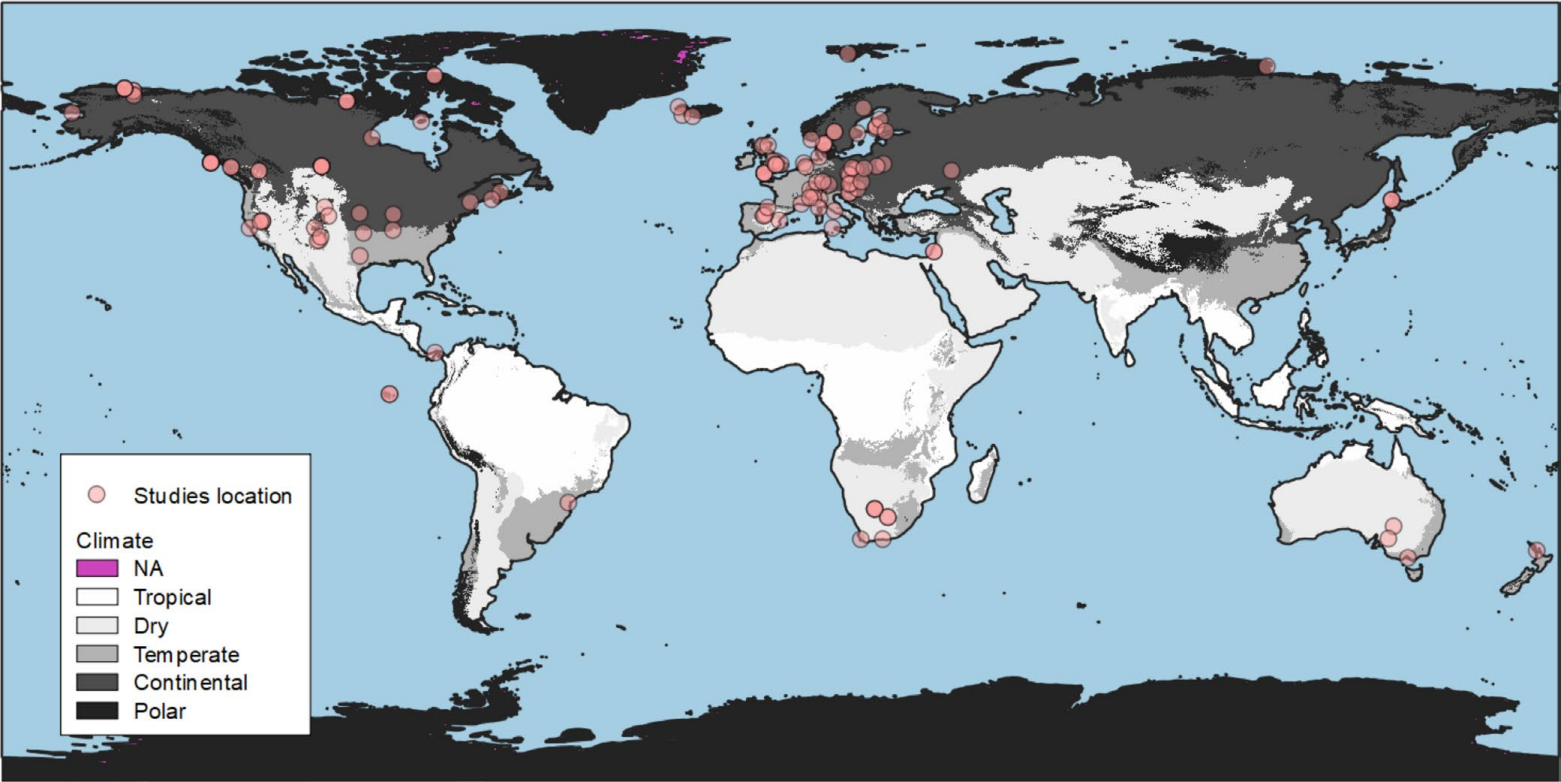
Global map of the study locations (*n* = 135). Symbols and colors represent the associated correlation sign between high air temperature and overall breeding success. The Köppen‐Geiger climate classification is represented in shades of gray, and studies location as dots. Location with multiple studies are represent as opaque dots.

The distribution of studies retained in our systematic review was uneven across the world, as previously observed in other articles (Figure 2; Cohen et al. 2018; Eyck et al. 2019). Most of the studies were conducted in North America or Europe. Our results are therefore based on a fraction of the globe, and some climates are over‐represented while others are under‐represented: continental (44.4%; *n* = 60/135), temperate (31.1%; *n* = 42/135), desert (13.3%; *n* = 18/135), polar (10.4%; *n* = 14/135), and tropical (0.7%; *n* = 1/135). Overall, 168 avian species and 17 orders were represented across all studies retained. Passeriformes were the most studied (51.5%; *n* = 118/229), followed by Charadriiformes (13.1%; *n* = 30/229), Anseriformes (10.0%; *n* = 23/229), Accipitriformes (5.2%; *n* = 12/229), Strigiformes (4.4%; *n* = 10/229), Galliformes (4.4%; *n* = 10/229), Falconiformes (3.1%; *n* = 7/229), and others (Piciformes, Bucerotiformes, Suliformes, Procellariiformes, Gruiformes, Columbiformes, Apodiformes, Psittaciformes, Cuculiformes, and Ciconiiformes; representing each < 3%). The high proportion of studies on Passeriformes is expected given that this order contains ~60% of all bird species (Jetz et al. 2012).

The proportion of studies investigating different breeding stages varied substantially, with most‐notably post‐hatching success being relatively over‐represented: breeding propensity (13.1%; *n* = 30/229), clutch size (36.2%; *n* = 83/229), hatching success (36.2%; *n* = 83/229), post‐hatching success (72.1%; *n* = 165/229), and post‐fledging success (10.5%; *n* = 24/229). Relatively few studies investigated breeding propensity and post‐fledging success, meaning that the influence of the thermal environment on early breeding investment and fledglings' fate may be under‐represented. Monitoring post‐fledging success requires a significant research effort, and depending on the species studied, it may be difficult to differentiate survival from dispersal when investigating variations in the recruitment rate (Coulson and Coulson 2008; Steenhof and Heath 2013; Wiebe 2020).

### 3.2 High Temperatures or Heat Stress?

The proportion of studies investigating hot events varied depending on the local climate, and arid climates were largely over‐represented (*n* = 12/18 studies; i.e., they represent 66.7% of the studies investigating hot events despite representing only 13.3% of all studies; Table 1). This observation may have two main explanations. First, authors may be more likely to describe extreme events as “heatwaves” or “extremely high temperature” in arid climates compared to cooler climates. This is supported by the fact that fewer studies test the effect of hot days or maximum temperatures in polar, continental, or temperate climates compared to arid climates (Table 1), suggesting that authors generally do not regard hot events as potential drivers of breeding success. Moreover, the direct effects of heat on breeding success were investigated only in a handful of studies (2.6% of the retained studies) and was mainly considered in arid climates (Table 2). Second, birds in cold climates may be less likely to suffer from heat stress during relatively hot events compared with birds in hotter climates. For instance, Dickey et al. (2008) described days with *T*_*a*_ > 0°C as “extremely high temperatures” in a polar environment, even though these conditions are unlikely to promote heat stress.

**TABLE 1:** Proportion of the studies investigating the effect of hot event and of different measures of temperatures on breeding success.

	Local climate				Overall (*n* = 229)
	Polar (*n* = 14)	Continental (*n* = 60)	Temperate (*n* = 42)	Arid (*n* = 18)	
Hot events*	14.3% (2/14)	8.3% (5/60)	9.5% (4/42)	66.7% (12/18)	14.9% (34/229)
Explanatory variable (T°)					
Hot days	7.1% (1/14)	8.3% (5/60)	1.7% (1/60)	27.8% (5/18)	7.9% (18/229)
Max *T*_*a*_	14.3% (2/14)	13.3% (8/60)	31.0% (13/42)	55.6% (10/18)	21.4% (49/229)
Mean *T*_*a*_	42.9% (6/14)	86.7% (52/60)	69.1% (29/42)	33.3% (6/18)	74.7% (171/229)
Min *T*_*a*_	7.1% (1/14)	15% (9/60)	23.8 (10/42)	16.7% (3/18)	14.9% (34/229)
*T*_*a*_ deviation	0% (0/14)	0% (0/60)	2.4% (1/42)	22.2% (4/18)	3.1% (7/229)
PCA	28.6% (4/14)	3.3% (2/60)	7.1% (3/42)	5.6% (1/18)	4.8% (11/229)
Cumulative *T*_*a*_	28.6% (4/14)	6.7% (4/60)	2.4% (1/42)	0% (0/18)	4.4% (10/229)

*Note:* *T*_*a*_: air temperature. *Hot events: *T*_*a*_ likely to promote heat stress (behavioral signs of heat dissipation, signs of dehydration or hyperthermia, *T*_*a*_ > UCT), hot days (e.g., 90th percentile of the hottest days) or based on authors description (e.g., “heatwave”). *T*_*a*_ deviation: deviation from the average temperature. Cumulative *T*_*a*_: sum of the temperature during a period.

**TABLE 2:** Proportion of the studies investigating the effect of temperature on breeding success considering heat stress, phenology, and trophic relationships.

	Local climate				Overall (*n* = 229)
	Polar (*n* = 14)	Continental (*n* = 60)	Temperate (*n* = 42)	Arid (*n* = 18)	
Heat stress	7.1% (1/14)	0% (0/60)	2.4% (1/42)	22.2% (4/18)	2.6% (6/229)
Phenology	85.7% (12/14)	81.7% (49/60)	85.7% (36/42)	50.0% (9/18)	70.7% (162/229)
Food	35.7% (5/14)	18.3% (11/60)	9.5% (4/42)	22.2% (4/18)	15.7% (36/229)
Predation	7.1% (1/14)	6.7% (4/60)	9.5% (4/42)	11% (2/18)	14.9% (34/229)
Competition	14.3% (2/14)	15% (9/60)	21.4% (9/42)	11% (2/18)	10.9% (25/229)

*Note:* Heat stress: behavioral signs of heat dissipation, signs of dehydration or hyperthermia. Competition: inter‐specific and intra‐specific competition.

Finally, most of the retained studies considered other mechanisms than temperature to be potential drivers of breeding success (i.e., phenology, food availability, predation or intra‐specific and inter‐specific competition; 75.6% of the studies; Table 2). It is worth noting that authors often only report statistics for the best performing models, and we were thus unable to quantify the proportion of studies testing the interaction between temperature and other drivers. Among indirect drivers, phenology was by far the most studied (70.7%), followed by food availability (15.7%), predation (14.9%), and competition (10.9%). Finally, the relatively few studies investigating trophic factors (i.e., food availability, predation or competition) highlight the need to study relationships between breeding success and weather effects at the ecosystem level.

## 4 Mechanisms Underlying the Relationship Between Temperature and Breeding Success

### 4.1 Direct Effects: Thermal Environment and Parental Care

#### 4.1.1 Heat Stress as a Driver of Breeding Success

**FIGURE 3:**
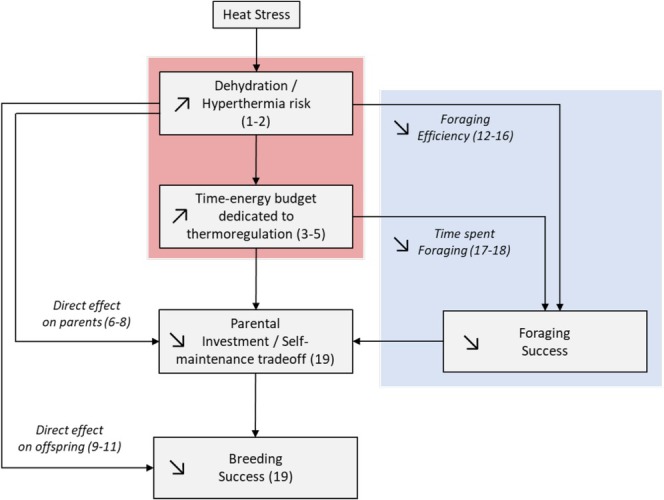
Schematic representation of the direct mechanisms (i.e., through thermoregulation) that mediates the link between heat stress and breeding success. The red and blue boxes correspond respectively to increased expenses and decreased resources. References: (1) Gerson et al. (2019) (2) McKechnie and Wolf (2019) (3) Smit et al. (2016) (4) Oswald et al. (2008) (5) Sharpe et al. (2021) (6) Moagi et al. (2021) (7) Bourne, Ridley, McKechnie, et al. (2021) (8) van de Ven et al. (2019) (9) Salaberria et al. (2014) (10) van de Ven et al. (2020) (11) Catry et al. (2015) (12) Danner et al. (2021) (13, 14) Tapper et al. (2020a, 2020b) (15) Oswald et al. (2021) (16) Barras et al. (2021) (17) Funghi et al. (2019) (18) Playà‐Montmany et al. (2023) (19) References within text.

Birds rely primarily on evaporative water loss for cooling and are subject to a trade‐off between dehydration and hyperthermia during heat exposure (Gerson et al. 2019; Smit et al. 2016). In warm temperate and arid biomes, there are several reports of wild birds showing signs of heat stress, such as thermoregulatory behaviors (e.g., panting, urohydrosis, shading; reviewed by McKechnie and Wolf 2019) and dehydration (Oswald et al. 2021; Salaberria et al. 2014; Sharpe et al. 2019; van de Ven et al. 2019, 2020). Moreover, exposure to high temperatures has been associated with physiological stress markers such as corticosterone levels (Moagi et al. 2021; Newberry and Swanson 2018) and the heterophils to lymphocyte ratio (H/L ratio; Catry et al. 2015; Skwarska et al. 2022). Finally, the activity pattern of breeding adults can be constrained in the heat, and parents may have to trade self‐maintenance and reproductive investment (Figure 3; AlRashidi et al. 2010; Amat and Masero 2004; Oswald et al. 2008).

Decreased breeding frequency and probability of second brooding have been reported during warm periods (e.g., Chambers, Gibbs, et al. 2008; Marques‐Santos et al. 2021; Mastrantonis et al. 2019). Clutch sizes were smaller when breeding season was reported to be warmer in several studies (e.g., Chambers, Quin, et al. 2008; Bowers et al. 2016; Londe et al. 2021). One explanation would be that high temperatures may hinder fertility (Hurley et al. 2018; Renthlei et al. 2021; Schou et al. 2021; for a review on poultry models see Vandana et al. 2021).

Hatching success decreased after exposure to high temperature in various studies (e.g., Dreitz et al. 2012; Duchardt et al. 2020; Grisham et al. 2016), including some reports of catastrophic egg loss (McCowan and Griffith 2021; Sharpe et al. 2021). Egg hatchability was most likely reduced by the direct effect of temperature on egg development and indirect effects mediated through parental care (e.g., Bourne, Ridley, McKechnie, et al. 2021; Clauser and McRae 2017; Sharpe et al. 2019). For example, signs of dehydration, reduced incubation consistencies, and decreased hatching success have been associated with high temperature in desert birds (Bourne, Ridley, McKechnie, et al. 2021; Sharpe et al. 2021). During extreme temperature events, adults have been reported to stop incubating and to show egg‐shading and heat dissipation behaviors, although it is unclear whether the function of such behaviors is to cool the adults or the eggs (Amat and Masero 2004; Brown and Downs 2003; Downs and Ward 1997; Sharpe et al. 2021). Exposure to such temperatures ultimately led to the desertion of the nest by the parents (Bourne, Ridley, McKechnie, et al. 2021; Sharpe et al. 2021), and nest desertion was related to the distance to a water source in Kentish Plover ( *Charadrius alexandrinus* ), highlighting a potential dehydration‐hyperthermia trade‐off (Amat and Masero 2004).

The same observations apply to post‐hatching success: both nestlings and parents can suffer from dehydration in the heat (Bourne, Ridley, Spottiswoode, et al. 2021; Oswald et al. 2021; Salaberria et al. 2014; van de Ven et al. 2020). During extreme events, nestlings might even be forced out of nests to avoid lethal temperatures (Catry et al. 2015). Some studies have shown a decreasing nestling provisioning rate at high temperatures (e.g., Barras et al. 2021; Oswald et al. 2021; Wiley and Ridley 2016), which could be linked with a diminished time spent foraging or foraging efficiency (e.g., Danner et al. 2021; Funghi et al. 2019; Playà‐Montmany et al. 2023). Individuals have to dedicate a greater proportion of time to thermoregulatory behaviors, and hyperthermia may constrain offspring provisioning even for temperate birds (Andreasson, Nilsson, and Nord 2020; Tapper et al. 2020a, 2020b). This could ultimately lead to a reduction in nestling growth and survival (Bourne, Ridley, Spottiswoode, et al. 2021; van de Ven et al. 2020). It is also worth noting that reduced growth when exposed to high temperatures can delay fledging and may increase the depredation probability (Cunningham, Martin, et al. 2013).

Thus, exposure to high temperatures can have lethal and sublethal effects on offspring phenotype (Conradie et al. 2019; Durant et al. 2013; Nord and Giroud 2020; Sauve et al. 2021). Given that growth is a strong predictor of post‐fledging survival (González‐Braojos et al. 2017; Maness and Anderson 2013; Rodríguez et al. 2016), suboptimal incubation, physiological stress, or reduced provisioning could contribute to a reduced offspring condition and post‐fledging survival. For instance, high temperatures experienced by offspring were related to decreased growth and reduced fledging and first‐year survival for the Southern pied babbler (Bourne et al. 2020a, 2020b). Importantly, early‐life development and thermal environment may influence life trajectories (Berntsen and Bech 2016; Hepp et al. 2015; Lindström 1999; Wada et al. 2015). This might have been the case for Gowshawk ( *Accipiter gentilis* ), which showed lower lifetime reproductive success when born during warmer springs (Herfindal et al. 2015).

#### 4.1.2 Thermal Relief as a Driver of Breeding Success

Cold exposure has been reported to impact avian reproduction in various populations from temperate to polar climates (e.g., Glądalski et al. 2020; Martin et al. 2017; Moreno et al. 2015; Tobolka et al. 2015; Winkler et al. 2013). Additionally, there are many reports of positive associations between high temperatures and breeding propensity (e.g., Väli 2012; Van Oudenhove et al. 2014), clutch size (e.g., Jónsson et al. 2009; Wright et al. 2009), hatching (e.g., Drever and Clark 2007; Mauck et al. 2017), post‐hatching (e.g., Kouba et al. 2020; Meller et al. 2018), and post‐fledging success in such systems (e.g., D'Alba et al. 2010; Layton‐Matthews et al. 2018). These studies suggest that high temperature may represent improved thermal conditions in some systems.

While avian embryo development occurs approximately in the 26°C–40°C range, it is thought to be optimal in much narrower ranges (Durant et al. 2013; Webb 1987). For instance, the optimal range of incubation temperature is ~35°C–37°C for Wood Ducks (*Aix sponsa*; Hepp et al. 2015). Since avian embryos produce very little heat and because optimal incubation temperature usually exceeds the environmental temperature, embryonic development is in most cases reliant on parental thermogenesis (Deeming and Reynolds 2015; Deeming 2004). Consequently, the energy expenditure of parents during incubation can be as high as during chick rearing (Williams and Vézina 2001). Additionally, nestlings reach thermo‐independence during post‐natal development, although the extent to which they are dependent on parental warming varies alongside the precocial‐altricial continuum (Ducatez and Field 2021; Price and Dzialowski 2017; Starck and Ricklefs 1998). Overall, breeding birds usually show increases in energy expenditure compared to non‐breeding individuals, and the energetic cost of incubation seems higher for arctic birds, suggesting an increased need for parental nest warming in cooler environments (Nord and Williams 2015; Williams 1996). High temperatures may consequently promote a thermal relief in some systems (i.e., decreased energy needed to maintain nest or body temperature through thermogenesis).

Parents energy expenditure during incubation has been reported to be minimal at high temperatures in several studies (De Heij et al. 2007; Haftorn and Reinertsen 1985; te Marvelde et al. 2012; Tulp et al. 2009). Moreover, there are many reports of decreased nest attentiveness and elongation of off‐bout duration with high temperatures in cold climates, suggesting an alleviated time investment of parents (e.g., Arct et al. 2022; Diez‐Mendez et al. 2021; Klimczuk et al. 2015; Williams and DeLeon 2020). This is further supported by studies reporting a decreased parental energy expenditure and nest attentiveness with experimental nest heating (Ardia et al. 2009; Bryan and Bryant 1999). For instance, the growth of Tree Swallow ( *Tachycineta bicolor* ) nestlings was positively influenced by experimental nest warming but negatively affected by nest cooling (Ardia et al. 2010; Pérez et al. 2008). Finally, in a subarctic environment, the growth of Dunlin ( *Calidris alpina* ) nestlings was maintained during a period of below‐average food availability only when temperature was high, suggesting a thermal relief (McKinnon et al. 2013). In summary, high temperatures may allow for a greater allocation to self‐maintenance of breeders, a higher breeding success, and/or better offspring quality (growth and physiological condition) in some systems.

### 4.2 Indirect Effects: Phenology and Trophic Interaction

#### 4.2.1 Phenology: Selection for Early Breeding, False Springs, and Breeding Season Duration

Bird phenology is plastic, and breeding timing has been reported to vary with environmental conditions. In most cases, breeding timing seems to advance with high air temperature (Phillimore et al. 2016; reviewed by Cohen et al. 2018; Radchuk et al. 2019). This phenomenon may be adaptive, as it allows individuals to breed when the conditions are the most suitable (Charmantier and Gienapp 2014; Charmantier et al. 2008; Lof et al. 2012), explaining the association between breeding dates and reproductive performance (reviewed by Dunn 2004; Dunn and Winkler 2010). Interestingly, the strength of the selection for earlier breeding seems to vary with environmental conditions (Kentie et al. 2018; Reed et al. 2009). For instance, the decreasing reproductive success along the breeding season, that is, for late breeders, was stronger during warmer springs in multiple studies (Bowers et al. 2016; Marrot et al. 2018; Whelan et al. 2017). Several studies reported prolonged or shortened breeding seasons with climate warming (Hällfors et al. 2020; Halupka and Halupka 2017; Møller et al. 2010). On one hand, high temperatures seem to favor early breeding, leading to an extended breeding season, an increased number of breeding attempts, and breeding success (Mingozzi et al. 2021). However, it may also be a misleading signal for the onset of breeding. Several studies reported negative effects of cold snaps on reproductive success after a warm early season, often referred to as the “false spring” phenomenon (e.g., Lehikoinen et al. 2009; Shipley et al. 2020; Skwarska et al. 2015). On the other hand, high temperatures in the late season may put an end to the breeding season (Jankowiak et al. 2014; Lv et al. 2020; Mares et al. 2017; Sharpe et al. 2021). For example, heatwaves shortened the Superb Fairy‐Wren ( *Malurus cyaneus* ) breeding season, which led to a reduced fledging success (Lv et al. 2020).

The influence of temperature on the timing of reproduction implies that it can also affect trophic interactions such as food availability, competition, predation, and parasitism (i.e., match‐mismatch hypothesis). The relationship between breeding success, temperature, these mechanisms, and their interaction with phenology is discussed below. We do not extensively review studies on this topic but intend to provide case studies highlighting the complexity of the relationship between high temperatures and breeding success.

#### 4.2.2 Trophic Interactions

##### 4.2.2.1 Food Availability

Temperature may drive breeding success through variation in food availability or quality, but multiple mechanisms may underlie this relationship (Barras et al. 2021; Pearce‐Higgins and Morris 2023). First, food availability may be directly dependent on temperature. High temperatures were associated with increased arthropod abundance and greater breeding success in continental and arctic biomes (Winkler et al. 2013; Ruthrauff et al. 2021). Winkler et al. (2013) even reported a similar temperature threshold for nestling mortality and insect availability, highlighting a strong association between them. On the contrary, arthropod abundance and activity decreased at high temperatures in arid climates (Holm and Edney 1973). This is not limited to insectivores, since avian species with various diets can also experience such effects on food availability (e.g., herbivorous or carnivorous; Doiron et al. 2015; Schmidt et al. 2020). The synchrony between maximum food abundance and breeding timing may differ during warm springs and may underlie influences of high temperatures on breeding success (i.e., “mismatch hypothesis”; Ross et al. 2017, 2018; Vatka et al. 2011, 2014, 2016; for a review see Visser et al. 2012). For instance, synchrony with caterpillar abundance increased during warmer seasons for the Willow Tit ( *Poecile montanus* ) in a boreal forest and was in turn positively correlated with nestling survival (Vatka et al. 2011). Lastly, temperature and breeding timing can both interact with abiotic factors. As reported by Ruthrauff et al. 2021, high temperatures correlated with early snowmelt, driving the arthropod emergence and thus promoting early breeding and a greater breeding success. For the Greater Snow Goose ( *Chen caerulescens atlantica* ), the mismatch between hatching date and peak nitrogen concentration in vegetation (an index of food availability and quality; Lepage et al. 1998) increased during warmer springs because of the early snowmelt, predicting in turn a reduced nestling growth (Doiron et al. 2015).

##### 4.2.2.2 Predation

Nest predation risk can be driven, either directly or indirectly, by the temperature through effects on activity patterns of birds, alternative prey, or predators. Birds can show reduced flight initiation distance when exposed to predators during heat events, suggesting a trade‐off between thermoregulation and predation risk (Gutiérrez et al. 2023). Predator activity can vary with temperature (Degregorio et al. 2014, 2015; Morris and Conner 2016), explaining an increased nest predation rate by snakes with high temperatures (e.g., D'Amelio et al. 2022; Oswald et al. 2020). Alternatively, high temperatures may also influence vegetation growth and depredation rate via changes in nest concealment (inhibit auditory, olfactory, and visual cues for predators; Borgmann et al. 2013). Finally, the number of prey available for the predators might vary in warm springs and therefore influence the predation risk (i.e., the alternative prey hypothesis; McKinnon et al. 2014). For example, predation on Eider Duck ( *Somateria mollissima* ) nests by polar bears increased when ice season was shortened (Iverson et al. 2014). Authors hypothesize that reduced ice coverage could hinder polar bears in their hunt for seals, leading to an increase in the rate of duck nest predation as an alternative prey.

##### 4.2.2.3 Parasitism

The performance of parasites is thought to vary with temperature in a curvilinear fashion (Aleuy and Kutz 2020; Ogden and Lindsay 2016). High temperature may increase the abundance of potential parasites in the nest (e.g., Branco et al. 2013; Prudhomme et al. 2015). Moreover, experimental heating of nests led to variation in the abundance of parasites (Dawson et al. 2005; Castaño‐Vázquez et al. 2018, 2021, 2022). For instance, the density of blowfly larvae in Tree Swallow (Ta*chycineta bicolor*) nests peaked at 25°C and decreased at both higher and lower temperatures (Dawson et al. 2005). The density of blowfly larvae was also more abundant around 23°C–25°C in Blue Tits (*Cyanistes caeruleus*) nests, with a strong decline below 20°C (Mennerat et al. 2021). As a result, increased nest parasitism associated with high temperatures can lead to reduced breeding success (Antoniazzi et al. 2011; Douglas and Pearce‐Higgins 2019; Møller 2010). Sublethal effects on nestling immunity (Dawson et al. 2005) adult and nestling body condition (Castaño‐Vázquez et al. 2021; Espinaze et al. 2020) may also hinder offspring survival later on.

##### 4.2.2.4 Competition

The intensity of the intra‐specific and inter‐specific competition may interact with the temperature, but to our knowledge, only a few studies seem to investigate it. However, there are reports of interactions between temperature, laying date, and competition on nest occupancy, clutch size, fledging success, and the probability of producing a recruit (Ahola et al. 2007, 2009, 2012; Bodey et al. 2021; Møller et al. 2020). Most notably, bird phenology may shift differently with temperature, which could lead to varying levels of competition during warm seasons (Ahola et al. 2007).

## 5 Summary

Our knowledge seems spatially confined to specific areas of the globe, mainly North America and Europe. It is crucial to increase research effort in overlooked areas and biomes, especially in tropical environments. Studies vary widely in their data acquisition and analysis, highlighting the need to establish guidelines for future studies. Most notably, the proportion of studies investigating hot events effects was considerably higher in arid environments. In such environments, authors may be more likely to use the terms “heatwaves”, “extreme events”, or “hot days”. High temperature is defined as relative to the species thermoregulation (i.e., likely to promote heat stress) in some studies and relative to the local temperature variability (e.g., hot days as 90th percentile of hottest days) in others. These definitions are, of course, not equivalent since a hot day does not necessarily promote heat stress and may even alleviate thermoregulatory costs depending on the system studied. Surprisingly, very few studies examined behavioral or physiological markers of heat exposure alongside reproductive success, resulting in limited insights into the potential occurrence of heat stress. Finally, multiple studies report complex and multifactorial relationships, with high temperature influencing breeding success through its interaction with phenology or trophic relationships. Overall, understanding and predicting the effects of high temperatures on avian breeding success require identifying the main drivers of reproductive performance, characterizing thermal constraints during reproduction, and evaluating their potential interactions. We hereafter present considerations for future studies.

## 6 Considerations for Future Studies

Potential effects of high temperature on avian breeding success and underlying mechanisms may vary depending on the local climate. High temperatures seem to be consistently linked with negative effects on desert bird reproduction (e.g., McCowan and Griffith 2021; Pattinson et al. 2022; Ridley et al. 2021), while results seem more mixed in cooler climates (e.g., Pipoly et al. 2013, 2022). This is supported by the intra‐specific variation in response to high temperature between different climates. For instance, the breeding success of the Barn Owl ( *Tyto alba* ) or the Mountain Plover ( *Charadrius montanus* ) was positively related to temperature in a temperate or continental climate, but negatively in a hotter semi‐arid climate (Barn Owl: Charter et al. 2017; Chausson et al. 2014, Mountain Plover: Dreitz et al. 2012; Pierce et al. 2019; Skagen and Adams 2012). Furthermore, while reproductive success has been positively associated with elevation in hot desert, a negative association is commonly reported in continental climates, suggesting different thermal constraints depending on the system studied (e.g., Hargrove et al. 2011; Nilsson et al. 2020).

Birds in hot environments may be more vulnerable to high temperature because their thermal environment is already close to their physiological limits, while birds in cool environments may benefit from it due to reduced energetic costs of keeping warm in otherwise cold conditions. For instance, energetic costs of thermoregulation are expected to decrease for Dovekies ( *Alle alle* ) in the arctic (Beaman et al. 2024), while desert species are expected to see their cooling costs increase with global warming (e.g., Albright et al. 2017; Conradie et al. 2020; McKechnie and Wolf 2010). This may be supported by the variation in thermal limits of birds such as the UCT. Although avian UCT has been reported to scales with the temperature in their habitat, it appears to be relatively conserved across species (Araújo et al. 2013; Qu and Wiens 2020; Song 2018; Sunday et al. 2019). For instance, in average, desert passerines seem to have a similar UCT as temperate passerines (desert: average UCT ~37.6°C across 30 species, McKechnie, Rushworth, et al. 2021; temperate: 37.7°C across 26 species, Cabello‐Vergel et al. 2022; Pollock et al. 2021; Data S3). Thus, desert birds may “persist near the edge of their physiological limit” (Iknayan and Beissinger 2018). Overall, hot events may be more likely to promote heat stress in deserts, and deleterious effects on desert birds survival, breeding success or demography are expected to increase with climate warming (Conradie et al. 2019; Riddell et al. 2019; Ridley et al. 2021).

Understanding the mechanisms underlying the association between high temperatures and breeding success requires a disentanglement of direct and indirect effects. Researchers should first consider the main drivers of breeding success within their system and investigate whether they interact with temperature. Since temperature usually increases during the breeding season, the association between temperature and reproductive output may be an artifact due to the breeding timing. Therefore, the correlation between breeding timing, reproductive success, and temperature should always be assessed. We strongly emphasize the need to measure markers of heat stress, either through behavior (e.g., panting, gular fluttering; McKechnie and Wolf 2019) or physiological markers (e.g., body temperature; Linek et al. 2021; plasma osmolarity or hematocrit for dehydration; Brischoux et al. 2020; Salaberria et al. 2014; Scope and Schwendenwein 2020), to pinpoint thermal constraints of breeding birds in their habitat. Experimental manipulation such as nest warming or cooling, food supplementation, or reducing predation represents important avenues for understanding relationships within a system (e.g., Corregidor‐Castro et al. 2023; D'Amelio et al. 2022). Multiple statistical procedures allow researchers to integrate hierarchical structures among predictors, and we encourage their use (e.g., Bourne et al. 2020a; Czeszczewik et al. 2020; van de Ven et al. 2020).

Considerations should be given to define relevant temperature measures before performing the data analysis to optimize the quality of the results. In some instances, the frequency with which the temperature exceeds a certain threshold has been reported to better predict the growth and survival of nestlings than maximum temperature (e.g., Conrey et al. 2016; Cunningham, Martin, et al. 2013). These thresholds can be defined based on the knowledge of the local climate (e.g., Mastrantonis et al. 2019; Pipoly et al. 2022; Smart et al. 2021), theoretical biological thresholds (e.g., McCowan and Griffith 2021) or empirical knowledge on species biology (e.g., Cunningham, Kruger, et al. 2013; Cunningham, Martin, et al. 2013). In addition, water availability most likely interacts with temperature, especially in arid systems where adequate water is necessary for survival and reproduction (Coe and Rotenberry 2003; Tieleman et al. 2004). In these systems, drought intensity is likely to drive breeding success (Bolger et al. 2005; Grisham et al. 2014; Lautenbach et al. 2018; Cox et al. 2020; Londe et al. 2021). Using an index of drought severity rather than temperature and precipitation on their own may be more relevant in these areas (e.g., Palmer Drought Severity Index; Cox et al. 2020). Overall, extreme temperature based on environmental stochasticity does not necessarily promote heat stress nor deleterious effects on reproduction. There is consequently a need to put local climate variability into perspective with meaningful biological thresholds (Cunningham, Kruger, et al. 2013). We strongly recommend systematically reporting temperature variables and summary statistics (e.g., mean temperature, average of maximum or minimum) and putting these measures in perspective with the local climate variability and the species thermoregulation.

Finally, species traits may influence the sensitivity of their reproductive output to high temperatures and represent relevant avenues for comparative studies. Numerous traits, such as thermal tolerance or breeding strategy, should be considered. For instance, drivers of breeding success and vulnerability to high temperatures may differ between migratory and sedentary species (Jones and Cresswell 2010; Jørgensen et al. 2016; Meller et al. 2018; Telenský et al. 2020), between single and multi‐brooded species (Møller et al. 2010; Halupka and Halupka 2017; Both et al. 2019; Halupka et al. 2023), and between single and bi‐parental or cooperative breeding species (Covas et al. 2008; Kosztolanyi et al. 2009; Jetz and Rubenstein 2011; Cornwallis et al. 2017; Bourne, Ridley, Spottiswoode, et al. 2021; D'Amelio et al. 2022). Passerines may represent relevant sentinel species since they are widespread and have limited heat tolerance compared to other avian taxa, explaining their over‐representation during heatwave‐induced mortality events (McKechnie, Gerson, et al. 2021).

## Author Contributions

**Adrien Levillain:** conceptualization (equal), data curation (lead), formal analysis (lead), investigation (lead), methodology (equal), supervision (supporting), validation (supporting), writing – original draft (lead), writing – review and editing (equal). **Sophie Reichert:** conceptualization (equal), funding acquisition (equal), methodology (equal), project administration (equal), supervision (equal), validation (equal), writing – review and editing (equal). **Sylvie Massemin:** conceptualization (equal), funding acquisition (equal), methodology (equal), project administration (equal), supervision (equal), validation (equal), writing – review and editing (equal).

## Conflicts of Interest

The authors declare no conflicts of interest.

## Supporting information


**Data S1.** Dataset of articles screened for eligibility.

**Data S2.** Dataset of all studies retained.

**Data S3.** Dataset of passerines UCT.

## Data Availability

Data are available as supporting information.

## References

[ece371771-bib-0001] Ahola, M. P. , T. Laaksonen , T. Eeva , and E. Lehikoinen . 2007. “Climate Change Can Alter Competitive Relationships Between Resident and Migratory Birds.” Journal of Animal Ecology 76: 1045–1052. 17922701 10.1111/j.1365-2656.2007.01294.x

[ece371771-bib-0002] Ahola, M. P. , T. Laaksonen , T. Eeva , and E. Lehikoinen . 2009. “Great Tits Lay Increasingly Smaller Clutches Than Selected for: A Study of Climate‐ and Density‐Related Changes in Reproductive Traits.” Journal of Animal Ecology 78: 1298–1306. 19682140 10.1111/j.1365-2656.2009.01596.x

[ece371771-bib-0003] Ahola, M. P. , T. Laaksonen , T. Eeva , and E. Lehikoinen . 2012. “Selection on Laying Date Is Connected to Breeding Density in the Pied Flycatcher.” Oecologia 168: 703–710. 21987266 10.1007/s00442-011-2135-5

[ece371771-bib-0004] Albright, T. P. , D. Mutiibwa , A. R. Gerson , et al. 2017. “Mapping Evaporative Water Loss in Desert Passerines Reveals an Expanding Threat of Lethal Dehydration.” Proceedings of the National Academy of Sciences of the United States of America 114: 2283–2288. 28193891 10.1073/pnas.1613625114 PMC5338552

[ece371771-bib-0005] Aleuy, O. A. , and S. Kutz . 2020. “Adaptations, Life‐History Traits and Ecological Mechanisms of Parasites to Survive Extremes and Environmental Unpredictability in the Face of Climate Change.” International Journal for Parasitology: Parasites and Wildlife 12: 308–317. 33101908 10.1016/j.ijppaw.2020.07.006 PMC7569736

[ece371771-bib-0006] AlRashidi, M. , A. Kosztolányi , C. Küpper , I. C. Cuthill , S. Javed , and T. Székely . 2010. “The Influence of a Hot Environment on Parental Cooperation of a Ground‐Nesting Shorebird, the Kentish Plover *Charadrius alexandrinus* .” Frontiers in Zoology 7: 1. 20148101 10.1186/1742-9994-7-1 PMC2819062

[ece371771-bib-0007] Amat, J. A. , and J. A. Masero . 2004. “How Kentish Plovers, *Charadrius alexandrinus* , Cope With Heat Stress During Incubation.” Behavioral Ecology and Sociobiology 56: 26–33.

[ece371771-bib-0008] Andreasson, F. , A. Hegemann , A. Nord , and J.‐Å. Nilsson . 2020. “Experimental Facilitation of Heat Loss Affects Work Rate and Innate Immune Function in a Breeding Passerine Bird.” Journal of Experimental Biology 223: jeb219790. 32179546 10.1242/jeb.219790

[ece371771-bib-0009] Andreasson, F. , J. Å. Nilsson , and A. Nord . 2020. “Avian Reproduction in a Warming World.” Frontiers in Ecology and Evolution 8: 576331.

[ece371771-bib-0010] Antoniazzi, L. R. , D. E. Manzoli , D. Rohrmann , M. J. Saravia , L. Silvestri , and P. M. Beldomenico . 2011. “Climate Variability Affects the Impact of Parasitic Flies on Argentinean Forest Birds.” Journal of Zoology 283: 126–134.

[ece371771-bib-0011] Araújo, M. B. , F. Ferri‐Yáñez , F. Bozinovic , P. A. Marquet , F. Valladares , and S. L. Chown . 2013. “Heat Freezes Niche Evolution.” Ecology Letters 16: 1206–1219. 23869696 10.1111/ele.12155

[ece371771-bib-0012] Arct, A. , R. Martyka , S. M. Drobniak , W. Oleś , A. Dubiec , and L. Gustafsson . 2022. “Effects of Elevated Nest Box Temperature on Incubation Behaviour and Offspring Fitness‐Related Traits in the Collared Flycatcher *Ficedula albicollis* .” Journal für Ornithologie 163: 263–272.

[ece371771-bib-0013] Ardia, D. R. , J. H. Pérez , E. K. Chad , M. A. Voss , and E. D. Clotfelter . 2009. “Temperature and Life History: Experimental Heating Leads Female Tree Swallows to Modulate Egg Temperature and Incubation Behaviour.” Journal of Animal Ecology 78: 4–13. 18637971 10.1111/j.1365-2656.2008.01453.x

[ece371771-bib-0014] Ardia, D. R. , J. H. Pérez , and E. D. Clotfelter . 2010. “Experimental Cooling During Incubation Leads to Reduced Innate Immunity and Body Condition in Nestling Tree Swallows.” Proceedings of the Royal Society B: Biological Sciences 277: 1881–1888. 10.1098/rspb.2009.2138 PMC2871872 20147326

[ece371771-bib-0015] Barras, A. G. , C. A. Niffenegger , I. Candolfi , Y. A. Hunziker , R. Arlettaz , and A. G. Barras . 2021. “Nestling Diet and Parental Food Provisioning in a Declining Mountain Passerine Reveal High Sensitivity to Climate Change.” Journal of Avian Biology 52: jav.02649.

[ece371771-bib-0016] Beaman, J. E. , C. R. White , M. Clairbaux , S. Perret , J. Fort , and D. Grémillet . 2024. “Cold Adaptation Does Not Handicap Warm Tolerance in the Most Abundant Arctic Seabird.” Proceedings of the Royal Society B: Biological Sciences 291: 20231887. 10.1098/rspb.2023.1887 PMC10791530 38228179

[ece371771-bib-0017] Beck, H. E. , N. E. Zimmermann , T. R. McVicar , N. Vergopolan , A. Berg , and E. F. Wood . 2018. “Present and Future Köppen‐Geiger Climate Classification Maps at 1‐Km Resolution.” Scientific Data 51: 1–12. 10.1038/sdata.2018.214 PMC6207062 30375988

[ece371771-bib-0018] Berntsen, H. H. , and C. Bech . 2016. “Incubation Temperature Influences Survival in a Small Passerine Bird.” Journal of Avian Biology 47: 141–145.

[ece371771-bib-0019] Bodey, T. W. , R. Barnett , C. R. du Feu , J. R. Clark , and S. Bearhop . 2021. “Nesting Outcomes Under Anthropogenic Change—Effects of Changing Climate and Nestbox Provision on the Reproduction of Great Tits *Parus major* .” Ibis 163, no. 1: 65–78.

[ece371771-bib-0020] Bolger, D. T. , M. A. Patten , and D. C. Bostock . 2005. “Avian Reproductive Failure in Response to an Extreme Climatic Event.” Oecologia 142: 398–406. 15549403 10.1007/s00442-004-1734-9

[ece371771-bib-0021] Borgmann, K. L. , C. J. Conway , and M. L. Morrison . 2013. “Breeding Phenology of Birds: Mechanisms Underlying Seasonal Declines in the Risk of Nest Predation.” PLoS One 8: e65909. 23776566 10.1371/journal.pone.0065909 PMC3680469

[ece371771-bib-0022] Both, C. , R. Ubels , and P. A. Ravussin . 2019. “Life‐History Innovation to Climate Change: Can Single‐Brooded Migrant Birds Become Multiple Breeders?” Journal of Avian Biology 50: 1–7.

[ece371771-bib-0023] Bourne, A. R. , S. J. Cunningham , C. N. Spottiswoode , and A. R. Ridley . 2020a. “High Temperatures Drive Offspring Mortality in a Cooperatively Breeding Bird.” Proceedings of the Royal Society B: Biological Sciences 287: 20201140. 10.1098/rspb.2020.1140 PMC7423658 33043866

[ece371771-bib-0024] Bourne, A. R. , S. J. Cunningham , C. N. Spottiswoode , and A. R. Ridley . 2020b. “Hot Droughts Compromise Interannual Survival Across all Group Sizes in a Cooperatively Breeding Bird.” Ecology Letters 23: 1776–1788. 32945068 10.1111/ele.13604

[ece371771-bib-0025] Bourne, A. R. , A. R. Ridley , A. E. McKechnie , C. N. Spottiswoode , and S. J. Cunningham . 2021. “Dehydration Risk Is Associated With Reduced Nest Attendance and Hatching Success in a Cooperatively Breeding Bird, the Southern Pied Babbler *Turdoides bicolor* .” Conservation Physiology 9: 1–16. 10.1093/conphys/coab043 PMC8208672 34150211

[ece371771-bib-0026] Bourne, A. R. , A. R. Ridley , C. N. Spottiswoode , and S. J. Cunningham . 2021. “Direct and Indirect Effects of High Temperatures on Fledging in a Cooperatively Breeding Bird.” Behavioral Ecology 32: 1212–1223.

[ece371771-bib-0027] Bowers, K. E. , J. L. Grindstaff , S. S. Soukup , et al. 2016. “Spring Temperatures Influence Selection on Breeding Date and the Potential for Phenological Mismatch in a Migratory Bird.” Ecology 97: 2880–2891. 27859132 10.1002/ecy.1516 PMC5119899

[ece371771-bib-0028] Boyles, J. G. , F. Seebacher , B. Smit , and A. E. McKechnie . 2011. “Adaptive Thermoregulation in Endotherms May Alter Responses to Climate Change.” Integrative and Comparative Biology 51: 676–690. 21690108 10.1093/icb/icr053

[ece371771-bib-0029] Branco, S. , C. Alves‐Pires , C. Maia , et al. 2013. “Entomological and Ecological Studies in a New Potential Zoonotic Leishmaniasis Focus in Torres Novas Municipality, Central Region, Portugal.” Acta Tropica 125: 339–348. 23262215 10.1016/j.actatropica.2012.12.008

[ece371771-bib-0030] Brischoux, F. , E. Beaugeard , B. Mohring , C. Parenteau , and F. Angelier . 2020. “Short‐Term Dehydration Influences Baseline but Not Stress‐Induced Corticosterone Levels in the House Sparrow (*Passer domesticus*).” Journal of Experimental Biology 223: 1–7. 10.1242/jeb.216424 31953365

[ece371771-bib-0031] Brown, M. , and C. T. Downs . 2003. “The Role of Shading Behaviour in the Thermoregulation of Breeding Crowned Plovers (*Vanellus coronatus*).” Journal of Thermal Biology 28: 51–58.

[ece371771-bib-0032] Bryan, S. M. , and D. M. Bryant . 1999. “Heating Nest‐Boxes Reveals an Energetic Constraint on Incubation Behaviour in Great Tits, *Parus major* .” Proceedings of the Royal Society of London. Series B: Biological Sciences 266: 157–162.

[ece371771-bib-0033] Cabello‐Vergel, J. , E. González‐Medina , M. Parejo , et al. 2022. “Heat Tolerance Limits of Mediterranean Songbirds and Their Current and Future Vulnerabilities to Temperature Extremes.” Journal of Experimental Biology 225: jeb244848. 36408945 10.1242/jeb.244848 PMC9789400

[ece371771-bib-0034] Castaño‐Vázquez, F. , J. Martínez , S. Merino , and M. Lozano . 2018. “Experimental Manipulation of Temperature Reduce Ectoparasites in Nests of Blue Tits *Cyanistes caeruleus* .” Journal of Avian Biology 49: e01695.

[ece371771-bib-0035] Castaño‐Vázquez, F. , S. Merino , F. Valera , and J. Veiga . 2022. “Experimental Manipulation of Humidity in a Cavity‐Nesting Bird Influences Ectoparasites' Abundance.” Parasitology 149, no. 4: 436–443. 35166204 10.1017/S0031182022000026 PMC10090615

[ece371771-bib-0036] Castaño‐Vázquez, F. , Y. R. Schumm , A. Bentele , P. Quillfeldt , and S. Merino . 2021. “Experimental Manipulation of Cavity Temperature Produces Differential Effects on Parasite Abundances in Blue Tit Nests at Two Different Latitudes.” International Journal for Parasitology: Parasites and Wildlife 14: 287–297. 33898230 10.1016/j.ijppaw.2021.03.010 PMC8056126

[ece371771-bib-0037] Catry, I. , T. Catry , P. Patto , A. M. A. Franco , and F. Moreira . 2015. “Differential Heat Tolerance in Nestlings Suggests Sympatric Species May Face Different Climate Change Risks.” Climate Research 66: 13–24.

[ece371771-bib-0038] Chambers, L. E. , H. Gibbs , M. A. Weston , and G. C. Ehmke . 2008. “Spatial and Temporal Variation in the Breeding of Masked Lapwings (*Vanellus miles*) in Australia.” Emu ‐ Austral Ornithology 108: 115–124.

[ece371771-bib-0039] Chambers, L. E. , B. R. Quin , P. Menkhorst , D. C. Franklin , and I. Smales . 2008. “The Effects of Climate on Breeding in the Helmeted Honeyeater.” Emu 108: 15–22.

[ece371771-bib-0040] Charmantier, A. , and P. Gienapp . 2014. “Climate Change and Timing of Avian Breeding and Migration: Evolutionary Versus Plastic Changes.” Evolutionary Applications 7: 15–28. 24454545 10.1111/eva.12126 PMC3894895

[ece371771-bib-0041] Charmantier, A. , R. H. Mccleery , L. R. Cole , C. Perrins , L. E. B. Kruuk , and B. C. Sheldon . 2008. “Adaptive Phenotypic Plasticity in Response to Climate Change in a Wild Bird Population.” Science 320: 800–803. 18467590 10.1126/science.1157174

[ece371771-bib-0042] Charter, M. , I. Izhaki , K. Meyrom , S. Aviel , Y. Leshem , and A. Roulin . 2017. “The Relationship Between Weather and Reproduction of the Barn Owl *Tyto alba* in a Semi‐Arid Agricultural Landscape in Israel.” Avian Biology Research 10: 2017–2253.

[ece371771-bib-0043] Chase, M. K. , N. Nur , and G. Geupel . 2005. “Effects of Weather and Population Density on Reproductive Success and Population Dynamics in a Song Sparrow (*Melospiza melodia*) Population: A Long‐Term Study.” Auk 122: 571–592.

[ece371771-bib-0044] Chausson, A. , I. Henry , B. Almasi , and A. Roulin . 2014. “Barn Owl ( *Tyto alba* ) Breeding Biology in Relation to Breeding Season Climate.” Journal für Ornithologie 155: 273–281.

[ece371771-bib-0045] Clauser, A. J. , and S. B. McRae . 2017. “Plasticity in Incubation Behavior and Shading by King Rails *Rallus elegans* in Response to Temperature.” Journal of Avian Biology 48: 479–488.

[ece371771-bib-0046] Clusella‐Trullas, S. , R. A. Garcia , J. S. Terblanche , and A. A. Hoffmann . 2021. “How Useful Are Thermal Vulnerability Indices?” Trends in Ecology & Evolution 36: 1000–1010. 34384645 10.1016/j.tree.2021.07.001

[ece371771-bib-0047] Coe, S. J. , and J. T. Rotenberry . 2003. “Water Availability Affects Clutch Size in a Desert Sparrow.” Ecology 84: 3240–3249.

[ece371771-bib-0048] Cohen, J. M. , M. J. Lajeunesse , and J. R. Rohr . 2018. “A Global Synthesis of Animal Phenological Responses to Climate Change.” Nature Climate Change 8: 224–228.

[ece371771-bib-0049] Conradie, S. R. , S. M. Woodborne , S. J. Cunningham , and A. E. McKechnie . 2019. “Chronic, Sublethal Effects of High Temperatures Will Cause Severe Declines in Southern African Arid‐Zone Birds During the 21st Century.” Proceedings of the National Academy of Sciences of the United States of America 116: 14065–14070. 31235571 10.1073/pnas.1821312116 PMC6628835

[ece371771-bib-0050] Conradie, S. R. , S. M. Woodborne , B. O. Wolf , A. Pessato , M. M. Mariette , and A. E. McKechnie . 2020. “Avian Mortality Risk During Heat Waves Will Increase Greatly in Arid Australia During the 21st Century.” Conservation Physiology 8: coaa048. 32523698 10.1093/conphys/coaa048 PMC7271765

[ece371771-bib-0051] Conrey, R. Y. , S. K. Skagen , A. A. Y. Adams , and A. O. Panjabi . 2016. “Extremes of Heat, Drought and Precipitation Depress Reproductive Performance in Shortgrass Prairie Passerines.” Ibis 158: 614–629.

[ece371771-bib-0052] Cornwallis, C. K. , C. A. Botero , D. R. Rubenstein , P. A. Downing , S. A. West , and A. S. Griffin . 2017. “Cooperation Facilitates the Colonization of Harsh Environments.” Nature Ecology & Evolution 1: 57. 28812731 10.1038/s41559-016-0057

[ece371771-bib-0053] Corregidor‐Castro, A. , J. Morinay , S. E. McKinlay , et al. 2023. “Experimental Nest Cooling Reveals Dramatic Effects of Heatwaves on Reproduction in a Mediterranean Bird of Prey.” Global Change Biology 29: 5552–5567. 37469036 10.1111/gcb.16888

[ece371771-bib-0054] Coulson, J. C. , and B. A. Coulson . 2008. “Measuring Immigration and Philopatry in Seabirds; Recruitment to Black‐Legged Kittiwake Colonies.” Ibis 150: 288–299.

[ece371771-bib-0055] Covas, R. , M. A. Du Plessis , and C. Doutrelant . 2008. “Helpers in Colonial Cooperatively Breeding Sociable Weavers *Philetairus socius* Contribute to Buffer the Effects of Adverse Breeding Conditions.” Behavioral Ecology and Sociobiology 63: 103–112.

[ece371771-bib-0056] Cox, W. A. , T. A. Dellinger , R. Kiltie , B. Bankovich , and B. Tornwall . 2020. “Factors Associated With Local and Statewide Population Trends of the Florida Sandhill Crane (Antigone Canadensis Pratensis).” Avian Conservation and Ecology 15: art7.

[ece371771-bib-0057] Cunningham, S. J. , J. L. Gardner , and R. O. Martin . 2021. “Opportunity Costs and the Response of Birds and Mammals to Climate Warming.” Frontiers in Ecology and the Environment 19: 300–307.

[ece371771-bib-0058] Cunningham, S. J. , A. C. Kruger , M. P. Nxumalo , and P. A. R. Hockey . 2013. “Identifying Biologically Meaningful Hot‐Weather Events Using Threshold Temperatures That Affect Life‐History.” PLoS One 8: e82492. 24349296 10.1371/journal.pone.0082492 PMC3861557

[ece371771-bib-0059] Cunningham, S. J. , R. O. Martin , C. L. Hojem , and P. A. R. Hockey . 2013. “Temperatures in Excess of Critical Thresholds Threaten Nestling Growth and Survival in a Rapidly‐Warming Arid Savanna: A Study of Common Fiscals.” PLoS One 8: e74613. 24040296 10.1371/journal.pone.0074613 PMC3767631

[ece371771-bib-0060] Czeszczewik, D. , P. Czortek , B. Jaroszewicz , K. Zub , P. Rowinski , and W. Walankiewicz . 2020. “Climate Change Has Cascading Effects on Tree Masting and the Breeding Performance of a Forest Songbird in a Primeval Forest.” Science of the Total Environment 747: 142084. 33076212 10.1016/j.scitotenv.2020.142084

[ece371771-bib-0061] D'Alba, L. , P. Monaghan , and R. G. Nager . 2010. “Advances in Laying Date and Increasing Population Size Suggest Positive Responses to Climate Change in Common Eiders *Somateria mollissima* in Iceland.” Ibis 152: 19–28.

[ece371771-bib-0062] D'Amelio, P. B. , A. C. Ferreira , R. Fortuna , et al. 2022. “Disentangling Climatic and Nest Predator Impact on Reproductive Output Reveals Adverse High‐Temperature Effects Regardless of Helper Number in an Arid‐Region Cooperative Bird.” Ecology Letters 25: 151–162. 34787354 10.1111/ele.13913 PMC9299450

[ece371771-bib-0063] Danner, R. M. , C. M. Coomes , and E. P. Derryberry . 2021. “Simulated Heat Waves Reduce Cognitive and Motor Performance of an Endotherm.” Ecology and Evolution 11: 2261–2272. 33717453 10.1002/ece3.7194 PMC7920763

[ece371771-bib-0064] Dawson, R. D. , K. K. Hillen , and T. L. Whitworth . 2005. “Effects of Experimental Variation in Temperature on Larval Densities of Parasitic Protocalliphora (Diptera: Calliphoridae) in Nests of Tree Swallows (Passeriformes: Hirundinidae).” Environmental Entomology 34: 563–568.

[ece371771-bib-0065] De Heij, M. E. , A. J. Van Der Graaf , D. Hafner , and J. M. Tinbergen . 2007. “Metabolic Rate of Nocturnal Incubation in Female Great Tits, *Parus major* , in Relation to Clutch Size Measured in a Natural Environment.” Journal of Experimental Biology 210: 2006–2012. 17515426 10.1242/jeb.001420

[ece371771-bib-0066] Deeming, D. C. 2004. Avian Incubation: Behaviour, Environment, and Evolution. Oxford University Press.

[ece371771-bib-0067] Deeming, D. C. , and D. J. Reynolds , eds. 2015. Nests, Eggs, and Incubation. Oxford University Press.

[ece371771-bib-0068] Degregorio, B. A. , S. J. Chiavacci , P. J. Weatherhead , J. D. Willson , T. J. Benson , and J. H. Sperry . 2014. “Snake Predation on North American Bird Nests: Culprits, Patterns and Future Directions.” Journal of Avian Biology 45: 325–333.

[ece371771-bib-0069] DeGregorio, B. A. , J. D. Westervelt , P. J. Weatherhead , and J. H. Sperry . 2015. “Indirect Effect of Climate Change: Shifts in Ratsnake Behavior Alter Intensity and Timing of Avian Nest Predation.” Ecological Modelling 312: 239–246.

[ece371771-bib-0070] Devictor, V. , R. Julliard , D. Couvet , and F. Jiguet . 2008. “Birds Are Tracking Climate Warming, but Not Fast Enough.” Proceedings of the Royal Society B: Biological Sciences 275: 2743–2748. 10.1098/rspb.2008.0878 PMC2605823 18713715

[ece371771-bib-0071] Dickey, M. H. , G. Gauthier , and M. C. Cadieux . 2008. “Climatic Effects on the Breeding Phenology and Reproductive Success of an Arctic‐Nesting Goose Species.” Global Change Biology 14: 1973–1985.

[ece371771-bib-0072] Diez‐Mendez, D. , C. B. Cooper , J. Jose Sanz , J. Verdejo , and E. Barba . 2021. “Deconstructing Incubation Behaviour in Response to Ambient Temperature Over Different Timescales.” Journal of Avian Biology 52: jav.02781.

[ece371771-bib-0073] Doiron, M. , G. Gauthier , and E. Lévesque . 2015. “Trophic Mismatch and Its Effects on the Growth of Young in an Arctic Herbivore.” Global Change Biology 21: 4364–4376. 26235037 10.1111/gcb.13057

[ece371771-bib-0074] Douglas, D. J. T. , and J. W. Pearce‐Higgins . 2019. “Variation in Ectoparasitic Sheep Tick *Ixodes ricinus* Infestation on European Golden Plover Chicks Pluvialis Apricaria and Implications for Growth and Survival.” Bird Study 66: 92–102.

[ece371771-bib-0075] Downs, C. T. , and D. Ward . 1997. “Does Shading Behavior of Incubating Shorebirds in Hot Environments Cool the Eggs or the Adults?” Auk 114: 717–724.

[ece371771-bib-0076] Dreitz, V. J. , R. Y. Conrey , and S. K. Skagen . 2012. “Drought and Cooler Temperatures Are Associated With Higher Nest Survival in Mountain Plovers.” Avian Conservation and Ecology 7: 7.

[ece371771-bib-0077] Drever, M. C. , and R. G. Clark . 2007. “Spring Temperature, Clutch Initiation Date and Duck Nest Success: A Test of the Mismatch Hypothesis.” Journal of Animal Ecology 76: 139–148. 17184362 10.1111/j.1365-2656.2006.01183.x

[ece371771-bib-0078] Du, W. G. , and R. Shine . 2015. “The Behavioural and Physiological Strategies of Bird and Reptile Embryos in Response to Unpredictable Variation in Nest Temperature.” Biological Reviews 90: 19–30. 24593133 10.1111/brv.12089

[ece371771-bib-0079] Ducatez, S. , and D. J. Field . 2021. “Disentangling the Avian Altricial‐Precocial Spectrum: Quantitative Assessment of Developmental Mode, Phylogenetic Signal, and Dimensionality.” Evolution 75: 2717–2735. 34608994 10.1111/evo.14365

[ece371771-bib-0080] Duchardt, C. J. , J. L. Beck , and D. J. Augustine . 2020. “Mountain Plover Habitat Selection and Nest Survival in Relation to Weather Variability and Spatial Attributes of Black‐Tailed Prairie Dog Disturbance.” Condor 122: 1–15.

[ece371771-bib-0081] Dunn, P. 2004. “Breeding Dates and Reproductive Performance.” In Advances in Ecological Research, edited by A. P. Moller , W. Fielder , and P. Berthold , 69–87. Elsevier.

[ece371771-bib-0082] Dunn, P. O. , and D. Winkler . 2010. “Effects of Climate Change on Timing of Breeding and Reproductive Success in Birds.” In Effect of Climate Change on Birds, 113–126. Oxford University Press.

[ece371771-bib-0083] Durant, S. E. , W. A. Hopkins , G. R. Hepp , and J. R. Walters . 2013. “Ecological, Evolutionary, and Conservation Implications of Incubation Temperature‐Dependent Phenotypes in Birds.” Biological Reviews 88: 499–509. 23368773 10.1111/brv.12015

[ece371771-bib-0084] Durant, S. E. , J. D. Willson , and R. B. Carroll . 2019. “Parental Effects and Climate Change: Will Avian Incubation Behavior Shield Embryos From Increasing Environmental Temperatures?” Integrative and Comparative Biology 59: 1068–1080. 31168619 10.1093/icb/icz083

[ece371771-bib-0085] Espinaze, M. P. A. , C. Hui , L. Waller , and S. Matthee . 2020. “Nest‐Type Associated Microclimatic Conditions as Potential Drivers of Ectoparasite Infestations in African Penguin Nests.” Parasitology Research 119: 3603–3616. 32996052 10.1007/s00436-020-06895-x

[ece371771-bib-0086] Eyck, H. J. F. , K. L. Buchanan , O. L. Crino , and T. S. Jessop . 2019. “Effects of Developmental Stress on Animal Phenotype and Performance: A Quantitative Review.” Biological Reviews 94: 1143–1160. 30609279 10.1111/brv.12496

[ece371771-bib-0087] Funghi, C. , L. S. C. McCowan , W. Schuett , and S. C. Griffith . 2019. “High Air Temperatures Induce Temporal, Spatial and Social Changes in the Foraging Behaviour of Wild Zebra Finches.” Animal Behaviour 149: 33–43.

[ece371771-bib-0088] Gerson, A. R. , A. E. McKechnie , B. Smit , et al. 2019. “The Functional Significance of Facultative Hyperthermia Varies With Body Size and Phylogeny in Birds.” Functional Ecology 33: 597–607.

[ece371771-bib-0089] Glądalski, M. , M. Bańbura , A. Kaliński , et al. 2020. “Extreme Temperature Drop Alters Hatching Delay, Reproductive Success, and Physiological Condition in Great Tits.” International Journal of Biometeorology 64: 623–629. 31915916 10.1007/s00484-019-01851-6

[ece371771-bib-0090] González‐Braojos, S. , J. J. Sanz , and J. Moreno . 2017. “Decline of a Montane Mediterranean Pied Flycatcher *Ficedula hypoleuca* Population in Relation to Climate.” Journal of Avian Biology 48: 1383–1393.

[ece371771-bib-0091] Grisham, B. A. , P. K. Borsdorf , C. W. Boal , and K. K. Boydston . 2014. “Nesting Ecology and Nest Survival of Lesser Prairie‐Chickens on the Southern High Plains of Texas.” Journal of Wildlife Management 78: 857–866.

[ece371771-bib-0092] Grisham, B. A. , A. J. Godar , C. W. Boal , and D. A. Haukos . 2016. “Interactive Effects Between Nest Microclimate and Nest Vegetation Structure Confirm Microclimate Thresholds for Lesser Prairie‐Chicken Nest Survival.” Condor 118: 728–746.

[ece371771-bib-0093] Gutiérrez, J. S. , T. Catry , M. Espinosa‐Colín , J. A. Masero , and J. P. Granadeiro . 2023. “Heat Stress Increases Risk Taking in Foraging Shorebirds.” Functional Ecology 37: 1005–1019.

[ece371771-bib-0094] Haftorn, S. , and R. E. Reinertsen . 1985. “The Effect of Temperature and Clutch Size on the Energetic Cost of Incubation in a Free‐Living Blue Tit (*Parus caeruleus*).” Auk 102: 470–478.

[ece371771-bib-0095] Hällfors, M. H. , L. H. Antaõ , M. Itter , et al. 2020. “Shifts in Timing and Duration of Breeding for 73 Boreal Bird Species Over Four Decades.” Proceedings of the National Academy of Sciences of the United States of America 117: 18557–18565. 32690693 10.1073/pnas.1913579117 PMC7414193

[ece371771-bib-0096] Halupka, L. , D. Arlt , J. Tolvanen , et al. 2023. “The Effect of Climate Change on Avian Offspring Production: A Global Meta‐Analysis.” Proceedings of the National Academy of Sciences of the United States of America 120: e2208389120. 37126701 10.1073/pnas.2208389120 PMC10175715

[ece371771-bib-0097] Halupka, L. , and K. Halupka . 2017. “The Effect of Climate Change on the Duration of Avian Breeding Seasons: A Meta‐Analysis.” Proceedings of the Royal Society B: Biological Sciences 284: 20171710. 10.1098/rspb.2017.1710 PMC5719171 29167360

[ece371771-bib-0098] Hargrove, L. , J. T. Rotenberry , L. Hargrove , and J. T. Rotenberry . 2011. “Breeding Success at the Range Margin of a Desert Species: Implications for a Climate‐Induced Elevational Shift.” Oikos 120: 1568–1576.

[ece371771-bib-0099] Hepp, G. R. , S. E. DuRant , and W. A. Hopkins . 2015. “Influence of Incubation Temperature on Offspring Phenotype and Fitness in Birds.” In Nests, Eggs, and Incubation, 171–178. Oxford University Press.

[ece371771-bib-0100] Herfindal, I. , M. van de Pol , J. T. Nielsen , B. E. Sæther , and A. P. Møller . 2015. “Climatic Conditions Cause Complex Patterns of Covariation Between Demographic Traits in a Long‐Lived Raptor.” Journal of Animal Ecology 84: 702–711. 25403010 10.1111/1365-2656.12318

[ece371771-bib-0101] Holm, E. , and E. B. Edney . 1973. “Daily Activity of Namib Desert Arthropods in Relation to Climate.” Ecology 54: 45–56.

[ece371771-bib-0102] Hurley, L. L. , C. S. McDiarmid , C. R. Friesen , S. C. Griffith , and M. Rowe . 2018. “Experimental Heatwaves Negatively Impact Sperm Quality in the Zebra Finch.” Proceedings of the Royal Society B: Biological Sciences 285: 20172547. 10.1098/rspb.2017.2547 PMC5805948 29343605

[ece371771-bib-0103] Iknayan, K. J. , and S. R. Beissinger . 2018. “Collapse of a Desert Bird Community Over the Past Century Driven by Climate Change.” Proceedings of the National Academy of Sciences of the United States of America 115: 8597–8602. 30082401 10.1073/pnas.1805123115 PMC6112692

[ece371771-bib-0104] Intergovernmental Panel on Climate Change . 2014. Climate Change 2014: Synthesis Report. IPCC.

[ece371771-bib-0105] Iverson, S. A. , H. G. Gilchrist , P. A. Smith , A. J. Gaston , and M. R. Forbes . 2014. “Longer Ice‐Free Seasons Increase the Risk of Nest Depredation by Polar Bears for Colonial Breeding Birds in the Canadian Arctic.” Proceedings of the Royal Society B: Biological Sciences 281: 20133128. 10.1098/rspb.2013.3128 PMC3924086 24500172

[ece371771-bib-0106] Jankowiak, L. , H. Pietruszewska , and D. Wysocki . 2014. “Weather Conditions and Breeding Season Length in Blackbird ( *Turdus merula* ).” Folia Zoologica 63: 245–250.

[ece371771-bib-0107] Jansen, D. Y. M. , R. Pradel , R. Mares , et al. 2019. “An Integrated Population Model Sheds Light on the Complex Population Dynamics of a Unique Colonial Breeder.” Population Ecology 61: 406–420.

[ece371771-bib-0108] Jenouvrier, S. , C. Barbraud , and H. Weimerskirch . 2003. “Effects of Climate Variability on the Temporal Population Dynamics of Southern Fulmars.” Journal of Animal Ecology 72: 576–587. 30893965 10.1046/j.1365-2656.2003.00727.x

[ece371771-bib-0109] Jetz, W. , and D. R. Rubenstein . 2011. “Environmental Uncertainty and the Global Biogeography of Cooperative Breeding in Birds.” Current Biology 21: 72–78. 21185192 10.1016/j.cub.2010.11.075

[ece371771-bib-0110] Jetz, W. , G. H. Thomas , J. B. Joy , K. Hartmann , and A. O. Mooers . 2012. “The Global Diversity of Birds in Space and Time.” Nature 491: 444–448. 23123857 10.1038/nature11631

[ece371771-bib-0111] Jones, T. , and W. Cresswell . 2010. “The Phenology Mismatch Hypothesis: Are Declines of Migrant Birds Linked to Uneven Global Climate Change?” Journal of Animal Ecology 79: 98–108. 19694874 10.1111/j.1365-2656.2009.01610.x

[ece371771-bib-0112] Jónsson, J. , A. Gardarsson , J. Gill , A. Petersen , and T. Gunnarsson . 2009. “Seasonal Weather Effects on the Common Eider, a Subarctic Capital Breeder, in Iceland Over 55 Years.” Climate Research 38: 237–248.

[ece371771-bib-0113] Jørgensen, P. S. , K. Böhning‐Gaese , K. Thorup , et al. 2016. “Continent‐Scale Global Change Attribution in European Birds—Combining Annual and Decadal Time Scales.” Global Change Biology 22: 530–543. 26486804 10.1111/gcb.13097

[ece371771-bib-0114] Kentie, R. , T. Coulson , J. C. E. W. Hooijmeijer , et al. 2018. “Warming Springs and Habitat Alteration Interact to Impact Timing of Breeding and Population Dynamics in a Migratory Bird.” Global Change Biology 24: 5292–5303. 30144224 10.1111/gcb.14406

[ece371771-bib-0115] Klimczuk, E. , L. Halupka , B. Czyz , M. Borowiec , J. J. Nowakowski , and H. Sztwiertnia . 2015. “Factors Driving Variation in Biparental Incubation Behaviour in the Reed Warbler *Acrocephalus scirpaceus* .” Ardea 103: 51–59.

[ece371771-bib-0116] Kosztolanyi, A. , S. Javed , C. Kupper , I. C. Cuthill , A. Al Shamsi , and T. Szekely . 2009. “Breeding Ecology of Kentish Plover *Charadrius alexandrinus* in an Extremely Hot Environment.” Bird Study 56: 244–252.

[ece371771-bib-0117] Kouba, M. , L. Bartoš , J. Bartošová , K. Hongisto , and E. Korpimäki . 2020. “Interactive Influences of Fluctuations of Main Food Resources and Climate Change on Long‐Term Population Decline of Tengmalm's Owls in the Boreal Forest.” Scientific Reports 10: 1–14. 33235236 10.1038/s41598-020-77531-y PMC7687899

[ece371771-bib-0118] Kubelka, V. , B. K. Sandercock , T. Székely , and R. P. Freckleton . 2022. “Animal Migration to Northern Latitudes: Environmental Changes and Increasing Threats.” Trends in Ecology & Evolution 37: 30–41. 34579979 10.1016/j.tree.2021.08.010

[ece371771-bib-0119] Lautenbach, J. M. , D. A. Haukos , D. S. Sullins , et al. 2018. “Factors Influencing Nesting Ecology of Lesser Prairie‐Chickens.” Journal of Wildlife Management 83: 205–215.

[ece371771-bib-0120] Layton‐Matthews, K. , A. Ozgul , and M. Griesser . 2018. “The Interacting Effects of Forestry and Climate Change on the Demography of a Group‐Living Bird Population.” Oecologia 186: 907–918. 29492692 10.1007/s00442-018-4100-z

[ece371771-bib-0121] Lehikoinen, A. , P. Byholm , E. Ranta , et al. 2009. “Reproduction of the Common Buzzard at Its Northern Range Margin Under Climatic Change.” Oikos 118: 829–836.

[ece371771-bib-0122] Lepage, D. , G. Gauthier , and A. Reed . 1998. “Seasonal Variation in Growth of Greater Snow Goose Goslings: The Role of Food Supply.” Oecologia 114: 226–235. 28307936 10.1007/s004420050440

[ece371771-bib-0123] Lindström, J. 1999. “Early Development and Fitness in Birds and Mammals.” Trends in Ecology & Evolution 14: 343–348. 10441307 10.1016/s0169-5347(99)01639-0

[ece371771-bib-0124] Linek, N. , T. Volkmer , J. R. Shipley , et al. 2021. “A Songbird Adjusts Its Heart Rate and Body Temperature in Response to Season and Fluctuating Daily Conditions.” Philosophical Transactions of the Royal Society, B: Biological Sciences 376: 20200213. 10.1098/rstb.2020.0213 PMC8200648 34121457

[ece371771-bib-0125] Lof, M. E. , T. E. Reed , J. M. McNamara , and M. E. Visser . 2012. “Timing in a Fluctuating Environment: Environmental Variability and Asymmetric Fitness Curves Can Lead to Adaptively Mismatched Avian Reproduction.” Proceedings of the Royal Society B: Biological Sciences 279: 3161–3169. 10.1098/rspb.2012.0431 PMC3385723 22628472

[ece371771-bib-0126] Londe, D. W. , R. D. Elmore , C. A. Davis , et al. 2021. “Weather Influences Multiple Components of Greater Prairie‐Chicken Reproduction.” Journal of Wildlife Management 85: 121–134.

[ece371771-bib-0127] Lv, L. , Y. Liu , H. L. Osmond , A. Cockburn , and L. E. B. Kruuk . 2020. “When to Start and When to Stop: Effects of Climate on Breeding in a Multi‐Brooded Songbird.” Global Change Biology 26: 443–457. 31581368 10.1111/gcb.14831

[ece371771-bib-0128] MacMillan, H. A. 2019. “Dissecting Cause From Consequence: A Systematic Approach to Thermal Limits.” Journal of Experimental Biology 222: jeb191593. 30796157 10.1242/jeb.191593

[ece371771-bib-0129] Mainwaring, M. C. , I. Barber , D. C. Deeming , D. A. Pike , E. A. Roznik , and I. R. Hartley . 2016. “Climate Change and Nesting Behaviour in Vertebrates: A Review of the Ecological Threats and Potential for Adaptive Responses.” Biological Reviews 92: 1991–2002. 27982504 10.1111/brv.12317

[ece371771-bib-0130] Maness, T. J. , and D. J. Anderson . 2013. “Predictors of Juvenile Survival in Birds.” Ornithological Monographs 78: 1–55.

[ece371771-bib-0131] Mares, R. , C. Doutrelant , M. Paquet , C. N. Spottiswoode , and R. Covas . 2017. “Breeding Decisions and Output Are Correlated With Both Temperature and Rainfall in an Arid‐Region Passerine, the Sociable Weaver.” Royal Society Open Science 4: 170835. 28989782 10.1098/rsos.170835 PMC5627122

[ece371771-bib-0132] Marques‐Santos, F. , U. Wischhoff , and M. Rodrigues . 2021. “Weather Fluctuations Are Linked to Nesting Success and Renesting Decisions in Saffron Finches.” Journal of Avian Biology 52: 1–12.

[ece371771-bib-0133] Marrot, P. , A. Charmantier , J. Blondel , and D. Garant . 2018. “Current Spring Warming as a Driver of Selection on Reproductive Timing in a Wild Passerine.” Journal of Animal Ecology 87: 754–764. 29337354 10.1111/1365-2656.12794 PMC5899892

[ece371771-bib-0134] Martin, K. , S. Wilson , E. C. Macdonald , A. F. Camfield , M. Martin , and S. A. Trefry . 2017. “Effects of Severe Weather on Reproduction for Sympatric Songbirds in an Alpine Environment: Interactions of Climate Extremes Influence Nesting Success.” Auk 134: 696–709.

[ece371771-bib-0135] Mastrantonis, S. , M. D. Craig , M. Renton , T. Kirkby , and R. J. Hobbs . 2019. “Climate Change Indirectly Reduces Breeding Frequency of a Mobile Species Through Changes in Food Availability.” Ecosphere 10: 1–11.

[ece371771-bib-0136] Mauck, R. A. , D. C. Dearborn , and C. E. Huntington . 2017. “Annual Global Mean Temperature Explains Reproductive Success in a Marine Vertebrate From 1955 to 2010.” Global Change Biology 24: 1599–1613. 29140586 10.1111/gcb.13982

[ece371771-bib-0137] Maxwell, S. L. , R. A. Fuller , T. M. Brooks , and J. E. M. Watson . 2016. “Biodiversity: The Ravages of Guns, Nets and Bulldozers.” Nature 536: 143–145. 27510207 10.1038/536143a

[ece371771-bib-0138] McCowan, L. S. C. , and S. C. Griffith . 2021. “Baked Eggs: Catastrophic Heatwave‐Induced Reproductive Failure in the Desert‐Adapted Zebra Finch (*Taeniopygia guttata*).” Ibis 163: 1207–1216.

[ece371771-bib-0139] McKechnie, A. E. , A. R. Gerson , and B. O. Wolf . 2021. “Thermoregulation in Desert Birds: Scaling and Phylogenetic Variation in Heat Tolerance and Evaporative Cooling.” Journal of Experimental Biology 224: jeb229211. 33627461 10.1242/jeb.229211

[ece371771-bib-0140] McKechnie, A. E. , I. A. Rushworth , F. Myburgh , and S. J. Cunningham . 2021. “Mortality Among Birds and Bats During an Extreme Heat Event in Eastern South Africa.” Austral Ecology 46: 687–691.

[ece371771-bib-0141] McKechnie, A. E. , and B. O. Wolf . 2010. “Climate Change Increases the Likelihood of Catastrophic Avian Mortality Events During Extreme Heat Waves.” Biology Letters 6: 253–256. 19793742 10.1098/rsbl.2009.0702 PMC2865035

[ece371771-bib-0142] McKechnie, A. E. , and B. O. Wolf . 2019. “The Physiology of Heat Tolerance in Small Endotherms.” Physiology 34: 302–313. 31389778 10.1152/physiol.00011.2019

[ece371771-bib-0143] McKinnon, L. , D. Berteaux , and J. Bêty . 2014. “Predator‐Mediated Interactions Between Lemmings and Shorebirds: A Test of the Alternative Prey Hypothesis.” Auk 131: 619–628.

[ece371771-bib-0144] McKinnon, L. , E. Nol , and C. Juillet . 2013. “Arctic‐Nesting Birds Find Physiological Relief in the Face of Trophic Constraints.” Scientific Reports 3: 1816. 23657421 10.1038/srep01816 PMC3648796

[ece371771-bib-0145] Meehl, G. A. , and C. Tebaldi . 2004. “More Intense, More Frequent, and Longer Lasting Heat Waves in the 21st Century.” Science 305: 994–997. 15310900 10.1126/science.1098704

[ece371771-bib-0146] Meller, K. , M. Piha , A. V. Vähätalo , and A. Lehikoinen . 2018. “A Positive Relationship Between Spring Temperature and Productivity in 20 Songbird Species in the Boreal Zone.” Oecologia 186: 883–893. 29350284 10.1007/s00442-017-4053-7

[ece371771-bib-0147] Mennerat, A. , A. Charmantier , P. Perret , S. Hurtrez‐Boussès , and M. M. Lambrechts . 2021. “Parasite Intensity Is Driven by Temperature in a Wild Bird.” Peer Community Journal 1: e60.

[ece371771-bib-0148] Milne, R. , S. J. Cunningham , A. T. K. Lee , and B. Smit . 2015. “The Role of Thermal Physiology in Recent Declines of Birds in a Biodiversity Hotspot.” Conservation Physiology 3: cov048. 27293732 10.1093/conphys/cov048 PMC4778484

[ece371771-bib-0149] Mingozzi, T. , P. Storino , G. Venuto , A. Massolo , and G. Tavecchia . 2021. “Climate Warming Induced a Stretch of the Breeding Season and an Increase of Second Clutches in a Passerine Breeding at Its Altitudinal Limits.” Current Zoology 68, no. 1: 9–17. 35169625 10.1093/cz/zoab029 PMC8836334

[ece371771-bib-0150] Mitchell, D. , S. K. Maloney , E. P. Snelling , V. de França Carvalho Fonsêca , and A. Fuller . 2024. “Measurement of Microclimates in a Warming World: Problems and Solutions.” Journal of Experimental Biology 227: jeb246481. 38958209 10.1242/jeb.246481

[ece371771-bib-0151] Mitchell, D. , E. P. Snelling , R. S. Hetem , S. K. Maloney , W. M. Strauss , and A. Fuller . 2018. “Revisiting Concepts of Thermal Physiology: Predicting Responses of Mammals to Climate Change.” Journal of Animal Ecology 87: 956–973. 29479693 10.1111/1365-2656.12818

[ece371771-bib-0152] Moagi, L. L. , A. R. Bourne , S. J. Cunningham , et al. 2021. “Hot Days Are Associated With Short‐Term Adrenocortical Responses in a Southern African Arid‐Zone Passerine Bird.” Journal of Experimental Biology 224: jeb242535. 34032270 10.1242/jeb.242535

[ece371771-bib-0153] Møller, A. P. 2010. “Host‐Parasite Interactions and Vectors in the Barn Swallow in Relation to Climate Change.” Global Change Biology 16: 1158–1170.

[ece371771-bib-0154] Møller, A. P. , J. Balbontín , A. A. Dhondt , et al. 2020. “Interaction of Climate Change With Effects of Conspecific and Heterospecific Density on Reproduction.” Oikos 129: 1807–1819.

[ece371771-bib-0155] Møller, A. P. , E. Flensted‐Jensen , K. Klarborg , W. Mardal , and J. T. Nielsen . 2010. “Climate Change Affects the Duration of the Reproductive Season in Birds.” Journal of Animal Ecology 79: 777–784. 20202013 10.1111/j.1365-2656.2010.01677.x

[ece371771-bib-0156] Moreno, J. , S. González‐Braojos , and R. Ruiz‐De‐Castañeda . 2015. “A Spring Cold Snap Is Followed by an Extreme Reproductive Failure Event in a Mountain Population of Pied Flycatchers *Ficedula hypoleuca* .” Bird Study 62: 466–473.

[ece371771-bib-0157] Morris, G. , and L. M. Conner . 2016. “Effects of Forest Management Practices, Weather, and Indices of Nest Predator Abundance on Nest Predation: A 12‐Year Artificial Nest Study.” Forest Ecology and Management 366: 23–31.

[ece371771-bib-0158] Neate‐Clegg, M. H. C. , B. A. Tonelli , and M. W. Tingley . 2024. “Advances in Breeding Phenology Outpace Latitudinal and Elevational Shifts for North American Birds Tracking Temperature.” Nature Ecology & Evolution 8: 2027–2036. 39223395 10.1038/s41559-024-02536-z

[ece371771-bib-0159] Newberry, G. N. , and D. L. Swanson . 2018. “Elevated Temperatures Are Associated With Stress in Rooftop‐Nesting Common Nighthawk (*Chordeiles minor*) Chicks.” Conservation Physiology 6: coy010. 29515805 10.1093/conphys/coy010 PMC5830973

[ece371771-bib-0160] Nilsson, A. L. K. , T. Reitan , T. Skaugen , et al. 2020. “Location Is Everything, but Climate Gets a Share: Analyzing Small‐Scale Environmental Influences on Breeding Success in the White‐Throated Dipper.” Frontiers in Ecology and Evolution 8: 306.

[ece371771-bib-0161] Nord, A. , and S. Giroud . 2020. “Lifelong Effects of Thermal Challenges During Development in Birds and Mammals.” Frontiers in Physiology 11: 1–9. 32523540 10.3389/fphys.2020.00419 PMC7261927

[ece371771-bib-0162] Nord, A. , and J. B. Williams . 2015. “The Energetic Costs of Incubation.” In Nests, Eggs, and Incubation, 152–170. Oxford University Press.

[ece371771-bib-0163] Ogden, N. H. , and L. R. Lindsay . 2016. “Effects of Climate and Climate Change on Vectors and Vector‐Borne Diseases: Ticks Are Different.” Trends in Parasitology 32: 646–656. 27260548 10.1016/j.pt.2016.04.015

[ece371771-bib-0164] Oswald, K. N. , E. F. Diener , J. P. Diener , S. J. Cunningham , B. Smit , and A. T. K. Lee . 2020. “Increasing Temperatures Increase the Risk of Reproductive Failure in a Near Threatened Alpine Ground‐Nesting Bird, the Cape Rockjumper *Chaetops frenatus* .” Ibis 162: 1363–1369.

[ece371771-bib-0165] Oswald, K. N. , B. Smit , A. T. K. Lee , C. L. Peng , C. Brock , and S. J. Cunningham . 2021. “Higher Temperatures Are Associated With Reduced Nestling Body Condition in a Range‐Restricted Mountain Bird.” Journal of Avian Biology 52: 1–10.

[ece371771-bib-0166] Oswald, S. A. , S. Bearhop , R. W. Furness , B. Huntley , and K. C. Hamer . 2008. “Heat Stress in a High‐Latitude Seabird: Effects of Temperature and Food Supply on Bathing and Nest Attendance of Great Skuas *Catharacta skua* .” Journal of Avian Biology 39: 163–169.

[ece371771-bib-0167] Pacifici, M. , C. Rondinini , J. R. Rhodes , et al. 2020. “Global Correlates of Range Contractions and Expansions in Terrestrial Mammals.” Nature Communications 11: 1–9. 10.1038/s41467-020-16684-w PMC7275054 32504033

[ece371771-bib-0168] Paniw, M. , T. D. James , C. Ruth Archer , et al. 2021. “The Myriad of Complex Demographic Responses of Terrestrial Mammals to Climate Change and Gaps of Knowledge: A Global Analysis.” Journal of Animal Ecology 90: 1398–1407. 33825186 10.1111/1365-2656.13467

[ece371771-bib-0169] Pattinson, N. B. , T. M. F. N. van de Ven , M. J. Finnie , L. J. Nupen , A. E. McKechnie , and S. J. Cunningham . 2022. “Collapse of Breeding Success in Desert‐Dwelling Hornbills Evident Within a Single Decade.” Frontiers in Ecology and Evolution 10: 842264.

[ece371771-bib-0170] Pearce‐Higgins, J. W. , and R. K. A. Morris . 2023. “Declines in Invertebrates and Birds—Could They Be Linked by Climate Change?” Bird Study 69: 59–71.

[ece371771-bib-0171] Pérez, J. H. , D. R. Ardia , E. K. Chad , and E. D. Clotfelter . 2008. “Experimental Heating Reveals Nest Temperature Affects Nestling Condition in Tree Swallows ( *Tachycineta bicolor* ).” Biology Letters 4: 468–471. 18628112 10.1098/rsbl.2008.0266 PMC2610083

[ece371771-bib-0172] Phillimore, A. B. , D. I. Leech , J. W. Pearce‐Higgins , and J. D. Hadfield . 2016. “Passerines May Be Sufficiently Plastic to Track Temperature‐Mediated Shifts in Optimum Lay Date.” Global Change Biology 22: 3259–3272. 27173755 10.1111/gcb.13302

[ece371771-bib-0173] Piatt, J. F. , J. K. Parrish , H. M. Renner , et al. 2020. “Extreme Mortality and Reproductive Failure of Common Murres Resulting From the Northeast Pacific Marine Heatwave of 2014–2016.” PLoS One 15: e0226087. 31940310 10.1371/journal.pone.0226087 PMC6961838

[ece371771-bib-0174] Pierce, A. K. , S. J. Dinsmore , and M. B. Wunder . 2019. “Decreased Nest Survival Associated With Low Temperatures in a High‐Elevation Population of Mountain Plover (*Charadrius montanus*).” Wilson Journal Of Ornithology 131: 502–513.

[ece371771-bib-0175] Pipoly, I. , V. Bókony , G. Seress , K. Szabó , and A. Liker . 2013. “Effects of Extreme Weather on Reproductive Success in a Temperate‐Breeding Songbird.” PLoS One 8: e80033. 24224033 10.1371/journal.pone.0080033 PMC3818280

[ece371771-bib-0176] Pipoly, I. , B. Preiszner , K. Sándor , et al. 2022. “Extreme Hot Weather Has Stronger Impacts on Avian Reproduction in Forests Than in Cities.” Frontiers in Ecology and Evolution 10: 218.

[ece371771-bib-0177] Playà‐Montmany, N. , E. González‐Medina , J. Cabello‐Vergel , et al. 2023. “Behavioural and Physiological Responses to Experimental Temperature Changes in a Long‐Billed and Long‐Legged Bird: A Role for Relative Appendage Size?” Behavioral Ecology and Sociobiology 77: 7.

[ece371771-bib-0178] Pollock, H. S. , J. D. Brawn , and Z. A. Cheviron . 2021. “Heat Tolerances of Temperate and Tropical Birds and Their Implications for Susceptibility to Climate Warming.” Functional Ecology 35: 93–104.

[ece371771-bib-0179] Price, E. R. , and E. M. Dzialowski . 2017. “Development of Endothermy in Birds: Patterns and Mechanisms.” Journal of Comparative Physiology B Biochemical, Systemic, and Environmental Physiology 188: 373–391. 29119278 10.1007/s00360-017-1135-0

[ece371771-bib-0180] Prudhomme, J. , N. Rahola , C. Toty , et al. 2015. “Ecology and Spatiotemporal Dynamics of Sandflies in the Mediterranean Languedoc Region (Roquedur Area, Gard, France).” Parasites and Vectors 8: 642. 26683841 10.1186/s13071-015-1250-2 PMC4684629

[ece371771-bib-0181] Qu, Y. F. , and J. J. Wiens . 2020. “Higher Temperatures Lower Rates of Physiological and Niche Evolution.” Proceedings of the Royal Society B: Biological Sciences 287: 20200823. 10.1098/rspb.2020.0823 PMC7423657 32673554

[ece371771-bib-0182] Radchuk, V. , T. Reed , C. Teplitsky , et al. 2019. “Adaptive Responses of Animals to Climate Change Are Most Likely Insufficient.” Nature Communications 10: 3109. 10.1038/s41467-019-10924-4 PMC6650445 31337752

[ece371771-bib-0183] Reed, T. E. , P. Warzybok , A. J. Wilson , R. W. Bradley , S. Wanless , and W. J. Sydeman . 2009. “Timing Is Everything: Flexible Phenology and Shifting Selection in a Colonial Seabird.” Journal of Animal Ecology 78: 376–387. 19054224 10.1111/j.1365-2656.2008.01503.x

[ece371771-bib-0184] Renthlei, Z. , L. Hmar , and A. Kumar Trivedi . 2021. “High Temperature Attenuates Testicular Responses in Tree Sparrow ( *Passer montanus* ).” General and Comparative Endocrinology 301: 113654. 33129830 10.1016/j.ygcen.2020.113654

[ece371771-bib-0185] Riddell, E. A. , K. J. Iknayan , B. O. Wolf , B. Sinervo , and S. R. Beissinger . 2019. “Cooling Requirements Fueled the Collapse of a Desert Bird Community From Climate Change.” Proceedings of the National Academy of Sciences of the United States of America 116: 21609–21615. 31570585 10.1073/pnas.1908791116 PMC6815107

[ece371771-bib-0186] Ridley, A. R. , E. M. Wiley , A. R. Bourne , S. J. Cunningham , and M. J. Nelson‐Flower . 2021. “Understanding the Potential Impact of Climate Change on the Behavior and Demography of Social Species: The Pied Babbler (*Turdoides bicolor*) as a Case Study.” In Advances in the Study of Behavior, 225–266. Academic Press Inc.

[ece371771-bib-0187] Rodríguez, S. , A. J. van Noordwijk , E. Álvarez , and E. Barba . 2016. “A Recipe for Postfledging Survival in Great Tits *Parus major*: Be Large and Be Early (But Not Too Much).” Ecology and Evolution 6: 4458–4467. 27386088 10.1002/ece3.2192 PMC4930993

[ece371771-bib-0188] Romano, M. D. , H. M. Renner , K. J. Kuletz , et al. 2020. “Die‐Offs, Reproductive Failure, and Changing At‐Sea Abundance of Murres in the Bering and Chukchi Seas in 2018.” Deep Sea Research Part II: Topical Studies in Oceanography 181: 104877.

[ece371771-bib-0189] Ross, M. V. , R. T. Alisauskas , D. C. Douglas , and D. K. Kellett . 2017. “Decadal Declines in Avian Herbivore Reproduction: Density‐Dependent Nutrition and Phenological Mismatch in the Arctic.” Ecology 98: 1869–1883. 28403519 10.1002/ecy.1856

[ece371771-bib-0190] Ross, M. V. , R. T. Alisauskas , D. C. Douglas , D. K. Kellett , and K. L. Drake . 2018. “Density‐Dependent and Phenological Mismatch Effects on Growth and Survival in Lesser Snow and Ross's Goslings.” Journal of Avian Biology 49: 1–12.

[ece371771-bib-0191] Ruthrauff, D. R. , V. P. Patil , J. W. Hupp , and D. H. Ward . 2021. “Life‐History Attributes of Arctic‐Breeding Birds Drive Uneven Responses to Environmental Variability Across Different Phases of the Reproductive Cycle.” Ecology and Evolution 11: 18514–18530. 35003689 10.1002/ece3.8448 PMC8717281

[ece371771-bib-0192] Salaberria, C. O. , P. Celis , I. L. Lopez‐rull , and D. Gil . 2014. “Effects of Temperature and Nest Heat Exposure on Nestling Growth, Dehydration and Survival in a Mediterranean Hole‐Nesting Passerine.” Ibis 156: 265–275.

[ece371771-bib-0193] Sauve, D. , V. L. Friesen , and A. Charmantier . 2021. “The Effects of Weather on Avian Growth and Implications for Adaptation to Climate Change.” Frontiers in Ecology and Evolution 9: 569741.

[ece371771-bib-0194] Schmidt, J. H. , J. Putera , and T. L. Wilson . 2020. “Direct and Indirect Effects of Temperature and Prey Abundance on Bald Eagle Reproductive Dynamics.” Oecologia 192: 391–401. 31858230 10.1007/s00442-019-04578-8

[ece371771-bib-0195] Schou, M. F. , M. Bonato , A. Engelbrecht , et al. 2021. “Extreme Temperatures Compromise Male and Female Fertility in a Large Desert Bird.” Nature Communications 12: 1–10. 10.1038/s41467-021-20937-7 PMC7854745 33531493

[ece371771-bib-0196] Scope, A. , and I. Schwendenwein . 2020. “Laboratory Evaluation of Renal Function in Birds.” Veterinary Clinics of North America: Exotic Animal Practice 23: 47–58. 31759451 10.1016/j.cvex.2019.08.002

[ece371771-bib-0197] Sharpe, L. , B. Cale , and J. L. Gardner . 2019. “Weighing the Cost: The Impact of Serial Heatwaves on Body Mass in a Small Australian Passerine.” Journal of Avian Biology 50: 1–9.

[ece371771-bib-0198] Sharpe, L. L. , C. Bayter , and J. L. Gardner . 2021. “Too Hot to Handle? Behavioural Plasticity During Incubation in a Small, Australian Passerine.” Journal of Thermal Biology 98: 102921. 34016345 10.1016/j.jtherbio.2021.102921

[ece371771-bib-0199] Shipley, J. R. , C. W. Twining , C. C. Taff , M. N. Vitousek , A. Flack , and D. W. Winkler . 2020. “Birds Advancing Lay Dates With Warming Springs Face Greater Risk of Chick Mortality.” Proceedings of the National Academy of Sciences of the United States of America 117: 25590–25594. 32989166 10.1073/pnas.2009864117 PMC7568286

[ece371771-bib-0200] Skagen, S. K. , and A. A. Y. Adams . 2012. “Weather Effects on Avian Breeding Performance and Implications of Climate Change.” Ecological Applications 22: 1131–1145. 22827123 10.1890/11-0291.1

[ece371771-bib-0201] Skwarska, J. , A. Kaliński , J. Wawrzyniak , et al. 2015. “Variation in Egg Sizes of Pied Flycatchers *Ficedula hypoleuca* in Central Poland: A Long‐Term Decreasing Trend.” Acta Ornithologica 50: 85–94.

[ece371771-bib-0202] Skwarska, J. , A. Podstawczyńska , M. Bańbura , et al. 2022. “Effects of Ambient Temperature During the Nestling Stage on a Stress Indicator in Nestling Pied Flycatchers *Ficedula hypoleuca* .” International Journal of Biometeorology 66: 139–148. 34618217 10.1007/s00484-021-02199-6 PMC8727405

[ece371771-bib-0203] Smart, Z. F. , M. G. Smith , and C. Riehl . 2021. “The El Niño—Southern Oscillation Dramatically Influences the Probability of Reproduction and Reproductive Rate of a Tropical Forest Bird.” Journal of Avian Biology 52: 1–12.

[ece371771-bib-0204] Smit, B. , G. Zietsman , R. O. Martin , S. J. Cunningham , A. E. McKechnie , and P. A. R. Hockey . 2016. “Behavioural Responses to Heat in Desert Birds: Implications for Predicting Vulnerability to Climate Warming.” Climatic Change Responses 3: 9.

[ece371771-bib-0205] Song, S. 2018. The Comparative Biology of Avian Thermoregulation at High Temperatures. Dr. Diss. UC Berkeley.

[ece371771-bib-0206] Starck, J. M. , and R. E. Ricklefs . 1998. “Patterns of Development: The Altricial‐Precocial Spectrum.” In Avian Growth and Development: Evolution in the Altricial Precocial Spectrum, 3–30. Oxford University Press.

[ece371771-bib-0207] Steenhof, K. , and J. A. Heath . 2013. “Local Recruitment and Natal Dispersal Distances of American Kestrels.” Condor 115: 584–592.

[ece371771-bib-0208] Stillman, J. H. 2019. “Heat Waves, the New Normal: Summertime Temperature Extremes Will Impact Animals, Ecosystems, and Human Communities.” Physiology 34: 86–100. 30724132 10.1152/physiol.00040.2018

[ece371771-bib-0209] Sunday, J. , J. M. Bennett , P. Calosi , et al. 2019. “Thermal Tolerance Patterns Across Latitude and Elevation.” Philosophical Transactions of the Royal Society, B: Biological Sciences 374: 20190036. 10.1098/rstb.2019.0036 PMC6606462 31203755

[ece371771-bib-0210] Tapper, S. , J. J. Nocera , and G. Burness . 2020a. “Experimental Evidence That Hyperthermia Limits Offspring Provisioning in a Temperate‐Breeding Bird.” Royal Society Open Science 7: 201589. 33204485 10.1098/rsos.201589 PMC7657879

[ece371771-bib-0211] Tapper, S. , J. J. Nocera , and G. Burness . 2020b. “Heat Dissipation Capacity Influences Reproductive Performance in an Aerial Insectivore.” Journal of Experimental Biology 223: jeb222232. 32321750 10.1242/jeb.222232

[ece371771-bib-0212] te Marvelde, L. , S. L. Webber , H. A. J. Meijer , and M. E. Visser . 2012. “Energy Expenditure During Egg Laying Is Equal for Early and Late Breeding Free‐Living Female Great Tits.” Oecologia 168: 631–638. 21935666 10.1007/s00442-011-2122-x PMC3277697

[ece371771-bib-0213] Telenský, T. , P. Klvaňa , M. Jelínek , J. Cepák , and J. Reif . 2020. “The Influence of Climate Variability on Demographic Rates of Avian Afro‐Palearctic Migrants.” Scientific Reports 10: 1–11. 33067507 10.1038/s41598-020-74658-w PMC7567877

[ece371771-bib-0214] Tieleman, B. I. , J. B. Williams , and G. Henk Visser . 2004. “Energy and Water Budget of Larks in a Life History Perspective: Parental Effort Varies With Aridity.” Ecology 85: 1399–1410.

[ece371771-bib-0215] Tobolka, M. , K. M. Zolnierowicz , and N. F. Reeve . 2015. “The Effect of Extreme Weather Events on Breeding Parameters of the White Stork *Ciconia ciconia* .” Bird Study 62: 377–385.

[ece371771-bib-0216] Tulp, I. , H. Schekkerman , L. W. Bruinzeel , J. Jukema , G. Henk Visser , and T. Piersma . 2009. “Energetic Demands During Incubation and Chick Rearing in a Uniparental and Biparental Shorebird Breeding in the High Arctic.” Auk 1: 155–164.

[ece371771-bib-0217] Ummenhofer, C. C. , and G. A. Meehl . 2017. “Extreme Weather and Climate Events With Ecological Relevance: A Review.” Philosophical Transactions of the Royal Society B 372: 20160135. 10.1098/rstb.2016.0135 PMC5434087 28483866

[ece371771-bib-0218] Väli, Ü. 2012. “Factors Limiting Reproductive Performance and Nestling Sex Ratio in the Lesser Spotted Eagle *Aquila pomarina* at the Northern Limit of Its Range: The Impact of Weather and Prey Abundance.” Acta Ornithologica 47: 157–168.

[ece371771-bib-0219] van de Ven, T. M. F. N. , A. E. McKechnie , and S. J. Cunningham . 2019. “The Costs of Keeping Cool: Behavioural Trade‐Offs Between Foraging and Thermoregulation Are Associated With Significant Mass Losses in an Arid‐Zone Bird.” Oecologia 191: 205–215. 31420741 10.1007/s00442-019-04486-x

[ece371771-bib-0220] van de Ven, T. M. F. N. , A. E. McKechnie , S. Er , and S. J. Cunningham . 2020. “High Temperatures Are Associated With Substantial Reductions in Breeding Success and Offspring Quality in an Arid‐Zone Bird.” Oecologia 193: 225–235. 32296953 10.1007/s00442-020-04644-6

[ece371771-bib-0221] Van Oudenhove, L. , G. Gauthier , and J. D. Lebreton . 2014. “Year‐Round Effects of Climate on Demographic Parameters of an Arctic‐Nesting Goose Species.” Journal of Animal Ecology 83: 1322–1333. 24724860 10.1111/1365-2656.12230

[ece371771-bib-0222] Vandana, G. D. , V. Sejian , A. M. Lees , P. Pragna , M. V. Silpa , and S. K. Maloney . 2021. “Heat Stress and Poultry Production: Impact and Amelioration.” International Journal of Biometeorology 65: 163–179. 33025116 10.1007/s00484-020-02023-7

[ece371771-bib-0224] Vatka, E. , M. Orell , and S. Rytkonen . 2016. “The Relevance of Food Peak Architecture in Trophic Interactions.” Global Change Biology 22: 1585–1594. 26527602 10.1111/gcb.13144

[ece371771-bib-0223] Vatka, E. , M. Orell , and S. Rytkönen . 2011. “Warming Climate Advances Breeding and Improves Synchrony of Food Demand and Food Availability in a Boreal Passerine.” Global Change Biology 17: 3002–3009.

[ece371771-bib-0225] Vatka, E. , S. Rytkönen , and M. Orell . 2014. “Does the Temporal Mismatch Hypothesis Match in Boreal Populations?” Oecologia 1: 595–605. 10.1007/s00442-014-3022-7 25024104

[ece371771-bib-0226] Visser, M. E. , L. te Marvelde , and M. E. Lof . 2012. “Adaptive Phenological Mismatches of Birds and Their Food in a Warming World.” Journal für Ornithologie 153: 75–84.

[ece371771-bib-0227] Wada, H. , B. Kriengwatana , N. Allen , K. L. Schmidt , K. K. Soma , and S. A. MacDougall‐Shackleton . 2015. “Transient and Permanent Effects of Suboptimal Incubation Temperatures on Growth, Metabolic Rate, Immune Function and Adrenocortical Responses in Zebra Finches.” Journal of Experimental Biology 218: 2847–2855. 26206355 10.1242/jeb.114108

[ece371771-bib-0228] Webb, D. R. 1987. “Thermal Tolerance of Avian Embryos: A Review.” Condor 89: 874–898.

[ece371771-bib-0229] Whelan, S. , D. Strickland , J. Morand‐Ferron , and D. R. Norris . 2017. “Reduced Reproductive Performance Associated With Warmer Ambient Temperatures During Incubation in a Winter‐Breeding, Food‐Storing Passerine.” Ecology and Evolution 7: 3029–3036. 28480002 10.1002/ece3.2864 PMC5415522

[ece371771-bib-0230] Wiebe, K. L. 2020. “Local Recruitment in Northern Flickers Is Related to Environmental Factors at Multiple Scales and Provides Reproductive Benefits to Yearling Breeders Settling Close to Home.” Auk 137: 1–10.

[ece371771-bib-0231] Wiley, E. M. , and A. R. Ridley . 2016. “The Effects of Temperature on Offspring Provisioning in a Cooperative Breeder.” Animal Behaviour 117: 187–195.

[ece371771-bib-0232] Williams, H. M. , and R. L. DeLeon . 2020. “Deep Learning Analysis of Nest Camera Video Recordings Reveals Temperature‐Sensitive Incubation Behavior in the Purple Martin (*Progne subis*).” Behavioral Ecology and Sociobiology 74: 7.

[ece371771-bib-0233] Williams, J. B. 1996. “Energetics of Avian Incubation.” In Avian Energetics and Nutritional Ecology, 375–415. Springer.

[ece371771-bib-0234] Williams, T. D. , and F. Vézina . 2001. “Reproductive Energy Expenditure, Intraspecific Variation and Fitness in Birds.” In Current Ornithology, 355–406. Springer US.

[ece371771-bib-0235] Winkler, D. W. , M. K. Luo , and E. Rakhimberdiev . 2013. “Temperature Effects on Food Supply and Chick Mortality in Tree Swallows ( *Tachycineta bicolor* ).” Oecologia 173: 129–138. 23468236 10.1007/s00442-013-2605-z PMC3751296

[ece371771-bib-0236] Wright, L. J. , R. A. Hoblyn , R. E. Green , et al. 2009. “Importance of Climatic and Environmental Change in the Demography of a Multi‐Brooded Passerine, the Woodlark *Lullula arborea* .” Journal of Animal Ecology 78: 1191–1202. 19594660 10.1111/j.1365-2656.2009.01582.x

